# Effect of a patient-centered hypertension delivery strategy on all-cause mortality: Secondary analysis of SEARCH, a community-randomized trial in rural Kenya and Uganda

**DOI:** 10.1371/journal.pmed.1003803

**Published:** 2021-09-20

**Authors:** Matthew D. Hickey, James Ayieko, Asiphas Owaraganise, Nicholas Sim, Laura B. Balzer, Jane Kabami, Mucunguzi Atukunda, Fredrick J. Opel, Erick Wafula, Marilyn Nyabuti, Lillian Brown, Gabriel Chamie, Vivek Jain, James Peng, Dalsone Kwarisiima, Carol S. Camlin, Edwin D. Charlebois, Craig R. Cohen, Elizabeth A. Bukusi, Moses R. Kamya, Maya L. Petersen, Diane V. Havlir

**Affiliations:** 1 Division of HIV, ID, & Global Medicine, Department of Medicine, UCSF, San Francisco, California, United States of America; 2 Centre for Microbiology Research, Kenya Medical Research Institute, Nairobi, Kenya; 3 Infectious Disease Research Collaboration, Kampala, Uganda; 4 School of Public Health, University of California Berkeley, Berkeley, California, United States of America; 5 School of Public Health & Health Sciences, University of Massachusetts Amherst, Amherst, Massachusetts, United States of America; 6 United States Agency for International Development, Kampala, Uganda; 7 Center for AIDS Prevention Studies & Department of Medicine, UCSF, San Francisco, California, United States of America; 8 Department of Obstetrics, Gynecology & Reproductive Sciences, UCSF, San Francisco, California, United States of America; 9 Department of Medicine, Makerere University, Kampala, Uganda; Harvard Medical School, UNITED STATES

## Abstract

**Background:**

Hypertension treatment reduces morbidity and mortality yet has not been broadly implemented in many low-resource settings, including sub-Saharan Africa (SSA). We hypothesized that a patient-centered integrated chronic disease model that included hypertension treatment and leveraged the HIV care system would reduce mortality among adults with uncontrolled hypertension in rural Kenya and Uganda.

**Methods and findings:**

This is a secondary analysis of the SEARCH trial (NCT:01864603), in which 32 communities underwent baseline population-based multidisease testing, including hypertension screening, and were randomized to standard country-guided treatment or to a patient-centered integrated chronic care model including treatment for hypertension, diabetes, and HIV. Patient-centered care included on-site introduction to clinic staff at screening, nursing triage to expedite visits, reduced visit frequency, flexible clinic hours, and a welcoming clinic environment. The analytic population included nonpregnant adults (≥18 years) with baseline uncontrolled hypertension (blood pressure ≥140/90 mm Hg). The primary outcome was 3-year all-cause mortality with comprehensive population-level assessment. Secondary outcomes included hypertension control assessed at a population level at year 3 (defined per country guidelines as at least 1 blood pressure measure <140/90 mm Hg on 3 repeated measures). Between-arm comparisons used cluster-level targeted maximum likelihood estimation.

Among 86,078 adults screened at study baseline (June 2013 to July 2014), 10,928 (13%) had uncontrolled hypertension. Median age was 53 years (25th to 75th percentile 40 to 66); 6,058 (55%) were female; 677 (6%) were HIV infected; and 477 (4%) had diabetes mellitus. Overall, 174 participants (3.2%) in the intervention group and 225 participants (4.1%) in the control group died during 3 years of follow-up (adjusted relative risk (aRR) 0.79, 95% confidence interval (CI) 0.64 to 0.97, *p* = 0.028). Among those with baseline grade 3 hypertension (≥180/110 mm Hg), 22 (4.9%) in the intervention group and 42 (7.9%) in the control group died during 3 years of follow-up (aRR 0.62, 95% CI 0.39 to 0.97, *p* = 0.038). Estimated population-level hypertension control at year 3 was 53% in intervention and 44% in control communities (aRR 1.22, 95% CI 1.12 to 1.33, *p* < 0.001). Study limitations include inability to identify specific causes of death and control conditions that exceeded current standard hypertension care.

**Conclusions:**

In this cluster randomized comparison where both arms received population-level hypertension screening, implementation of a patient-centered hypertension care model was associated with a 21% reduction in all-cause mortality and a 22% improvement in hypertension control compared to standard care among adults with baseline uncontrolled hypertension. Patient-centered chronic care programs for HIV can be leveraged to reduce the overall burden of cardiovascular mortality in SSA.

**Trial registration:**

ClinicalTrials.gov NCT01864603.

## Abstract 2

Matthew Hickey and co-workers report on outcomes of the SEARCH trial of hypertension screening and care in sub-Saharan Africa.

## Author summary

**Why was this study done?:**

- There is a growing burden of hypertension and resultant cardiovascular disease (CVD) globally, and particularly in sub-Saharan Africa (SSA).
- Maintenance of normal blood pressure through treatment with antihypertensive medications clearly reduces CVD morbidity and mortality; yet across SSA, only 28% are aware of their hypertension diagnosis, only 18% are on treatment, and only 7% have controlled blood pressure.
- The HIV chronic care system offers a platform for integrating noncommunicable disease (NCD) care for both people with HIV and the general population, though there are limited data on the implementation and effectiveness of such care models.

**What did the researchers do and find?:**

- We conducted a secondary analysis of data from the SEARCH cluster randomized controlled trial in 32 communities in Kenya and Uganda where all communities received population-wide hypertension and multidisease screening and were randomized to standard country-guided treatment or to a patient-centered integrated chronic care model including treatment for hypertension, diabetes, and HIV.
- Among 86,078 nonpregnant adults screened at baseline, we identified 10,928 (13%) with uncontrolled hypertension, all of whom were referred to the nearest government-run health facility for hypertension care.
- We conducted comprehensive vital status assessment after 3 years, determining vital status for 99% of participants. Among adults with baseline uncontrolled hypertension, all-cause mortality was 21% lower in intervention compared to control communities (3.1% versus 4.2%).

**What do these findings mean?:**

- Providing hypertension care through an integrated, patient-centered chronic care model was associated with a 21% reduction in all-cause mortality over 3 years among patients with uncontrolled hypertension at study baseline.
- Though the intervention was associated with improved hypertension control, half of participants remained uncontrolled, attenuating the benefits of hypertension treatment. Strategies to improve linkage and engagement in hypertension care are likely to further improve treatment outcomes in the context of integrated patient-centered hypertension care models.
- Leveraging the HIV care platform is a promising strategy for delivering chronic hypertension and other NCD care to address the growing burden of NCDs in SSA.

## Introduction

Hypertension is the most important risk factor for cardiovascular disease (CVD) and accounts for 14% of global mortality [1]. In sub-Saharan Africa (SSA), the prevalence of hypertension has been steadily increasing [2,3], contributing to a significant rise in CVD-associated mortality [4]. Despite rising prevalence in SSA, population-based surveys demonstrate substantial inequity in hypertension control compared to other regions [5], with only one-quarter of adults with hypertension aware of their diagnosis, 18% receiving treatment, and 7% achieving hypertension control [6]. Although there is a strong evidence base that hypertension treatment decreases the incidence of CVD and reduces mortality [7,8], this evidence has not been translated into practice in SSA.

In contrast to limited implementation of hypertension treatment, the HIV response has created a broad chronic care delivery system across much of SSA, providing a clear opportunity to leverage this infrastructure for other high-burden chronic diseases [9,10]. Several studies have evaluated interventions to improve integration of hypertension treatment with HIV care; however, these approaches have had limited impact on hypertension treatment outcomes [11–16].

The Sustainable East Africa Research in Community Health (SEARCH) study was a cluster randomized trial in Kenya and Uganda designed to evaluate a universal test and treat strategy on HIV incidence and community health outcomes [17]. SEARCH conducted population-level screening for hypertension and HIV at study baseline, reaching 90% of the census-enumerated adult population for HIV testing and 70% for hypertension screening [17,18]. In intervention communities, SEARCH implemented a patient-centered, streamlined care delivery model for integrated noncommunicable disease (NCD) and HIV care [19,20]. Previously published analyses of prespecfied trial outcomes demonstrated that this community-level intervention reduced HIV-associated mortality and improved population-level viral suppression [17]. In this analysis, our objective was to evaluate the effect of the SEARCH patient-centered, streamlined care intervention on mortality and hypertension care cascade outcomes among adults with uncontrolled hypertension identified at baseline population-level screening.

## Methods

### Study setting and population

The SEARCH study (NCT:01864603) was a pair-matched cluster randomized controlled trial in 32 rural communities Kenya and Uganda conducted over 3 years from 2013 to 2017 [17]. At baseline, we conducted a community-wide census to enumerate residents in each of the 32 communities, followed by 2-week community health campaigns that offered screening for HIV, hypertension, and diabetes [21]. Community health campaigns were held again in all communities after 3 years of follow-up and annually in intervention communities. This study is reported according to the Consort Statement for cluster randomized trials (S1 Consort Checklist).

At community health campaigns, hypertension screening was conducted using an algorithm based on WHO/International Society of Hypertension guidelines (S1 File) [22]. Using an electronic sphygmomanometer, all adults ≥18 years had a single blood pressure measurement. Those with an elevated initial blood pressure (systolic ≥140 mm Hg or diastolic ≥90 mm Hg) had 2 repeat measurements separated by at least 1 minute of rest. A diagnosis of uncontrolled hypertension was made if systolic blood pressure was ≥140 mm Hg or diastolic blood pressure ≥90 mm Hg on all measurements, per contemporary WHO guidelines [22]. Diabetes screening was also conducted among adults with risk factors or symptoms of hyperglycemia using random fingerstick glucose measurement; diagnosis was based on random glucose >11 mmol/L or self-reported history of diabetes (S2 File). Our population for this study included nonpregnant adults (age ≥18 years) with uncontrolled hypertension identified through baseline screening.

### Linkage to care

In all 32 communities, participants diagnosed with uncontrolled hypertension at baseline were scheduled for an appointment at the nearest government-run health center within each community. Individuals with comorbid HIV infection additionally received a 1-time transportation voucher to facilitate linkage to care. In intervention communities, additional strategies were implemented to facilitate linkage. First, at community health campaigns, patients were introduced to a clinic staff member, in person or by phone, and were given a phone number to call with questions. Second, individuals with comorbid HIV infection who missed their initial clinic appointment received a phone call or, if the phone call was unsuccessful, a home visit to reschedule their appointment [23].

### Streamlined hypertension care intervention

The SEARCH patient-centered streamlined care model focused on reducing structural barriers to care and improving relationships between patients and clinic, as described previously [19,20]. Hypertension, diabetes, and HIV treatment were provided using an integrated care model, which featured a nurse-driven triage system to tailor visits based on patient need and reduce waiting time, flexible clinic hours, phone-based appointment reminders, telephone access to clinicians, and a welcoming environment with clinicians and staff trained on providing friendly services. Blood pressure medications were prescribed for 12 weeks when hypertension was controlled (<140/90 mm Hg) and for 4 weeks when uncontrolled, using standardized protocols adapted from national guidelines (S1 File) [19]. Treatment for those with comorbid diabetes was also provided using a country guideline-based algorithm (S2 File). Clinic medication supplies were supplemented by the study, and medications were provided free of charge. Clinicians received initial training on treatment algorithms and streamlined care, followed by refresher trainings and on-site mentoring approximately 3 to 4 times per year during the study. Both HIV-uninfected and HIV-infected individuals received care using the same streamlined model. For people with HIV and hypertension, care was delivered during the same visit, with both HIV and hypertension medications provided during clinical consultation.

### Control hypertension care

In control communities, NCD care was provided at the general outpatient department in larger clinics and at the HIV clinic at smaller clinics. To reduce the possibility that differential clinic staffing would be the primary driver of outcomes, clinics in control communities were provided with similar staffing to that of intervention clinics, generally a clinical officer and nurse at each clinic. Hypertension and diabetes treatment was administered using the same treatment guidelines as in the intervention; clinic medication supplies were supplemented by the study, and medications were provided free of charge. Clinicial staff received initial training on treatment algorithms and referesher trainings approximately twice per year during the study.

### Outcome measurement and definition

Vital status was assessed during a repeat community health campaign conducted in each community at year 3. Individuals who were not seen at year 3 community health campaigns were tracked to ascertain vital status from the individual or via interview with a household member, neighbor, or community leader [17].

At baseline and year 3, hypertension was classified according to the lowest measured blood pressure, described above, as controlled (<140/90 mm Hg), grade 1 (140 to 159/90 to 99 mm Hg), grade 2 (160 to 179/100 to 109 mm Hg), or grade 3 (≥180/110 mm Hg) [22]. Since participation in community health campaigns was independent from linkage and retention in care, blood pressure assessment in this study did not rely on clinic measures.

We defined linkage to hypertension care as ≥1 clinic visit during the first year of follow-up and engagement in hypertension care as ≥1 clinic visit during each year of follow-up.

### Statistical analysis

This was a secondary analysis of prespecified outcomes in the SEARCH trial (mortality, hypertension control, linkage, and engagement) among persons with baseline uncontrolled hypertension. Statistical analyses were conducted using a prespecified analysis plan (S1 Statistical Analysis Plan). As previously detailed [17,24], sample size and power calculations were conducted for the trial’s primary outcome, HIV incidence, and communities were randomized to the intervention or control condition within matched pairs.

We evaluated the effect of the SEARCH intervention on 3-year mortality among nonpregnant adults (age ≥18 years) with uncontrolled hypertension identified at baseline population-level screening. To do so, we compared mortality by follow-up year 3 between intervention and control communities using a 2-staged approach, accounting for clustering and the matched-pair design [17,24]. We first calculated the proportion of participants in each community who died by year 3, excluding persons whose year 3 vital status was unknown. We then compared mortality between study arms using community-level targeted maximum likelihood estimation (TMLE) with cross-validation to select baseline adjustment variables from the following prespecified set: the proportion of participants aged ≥60 years and the proportion of participants with grade 2 or greater hypertension severity [25]. In sensitivity analyses, we excluded persons aged ≥80 years given that hypertension treatment may be deferred in some elderly individuals, and persons with baseline HIV infection to isolate the effect of improved hypertension care from effects due to improvement in HIV care.

We used a similar 2-stage procedure to evaluate the effect of the intervention on population-level hypertension control at year 3 (lowest blood pressure <140/90 mm Hg), using TMLE to adjust for differences in characteristics between persons with measurements and persons with missing measurements [17,24,26]. Additionally, we reported changes in grade of hypertension severity and median reduction in systolic blood pressure between baseline and year 3.

To understand intervention impacts on distinct components of the hypertension care cascade, we compared the proportions who attained each step in the hypertension care cascade (linkage, engagement, and hypertension control) at year 3 using the 2-stage approach. Due to limitations of clinic visit data for hypertension care in Kenya, this analysis included only the 10 intervention and 10 control communities in Uganda.

To understand heterogeneity in intervention effects, we repeated analyses of each outcome stratifying on sex and baseline hypertension severity. We also conducted post hoc stratification of results by country.

Finally, to understand the risk factors for mortality, hypertension control, and linkage to care among adults with baseline uncontrolled hypertension, we assessed the following individual-level predictors: age, sex, baseline hypertension severity, body mass index (BMI) category, HIV status, and diabetes status. We used TMLE to obtain adjusted relative risks (aRRs) for each predictor accounting for clustering by community.

### Ethical considerations

The study was approved by the Makerere University School of Medicine Research and Ethics Committee, the Uganda National Council for Science and Technology, the Kenya Medical Research Instittue Ethical Review Committee, and the University of California San Francisco Committee on Human Research. Verbal informed consent was obtained for all participants at study enrollment.

## Results

### Study population

**Fig 1:**
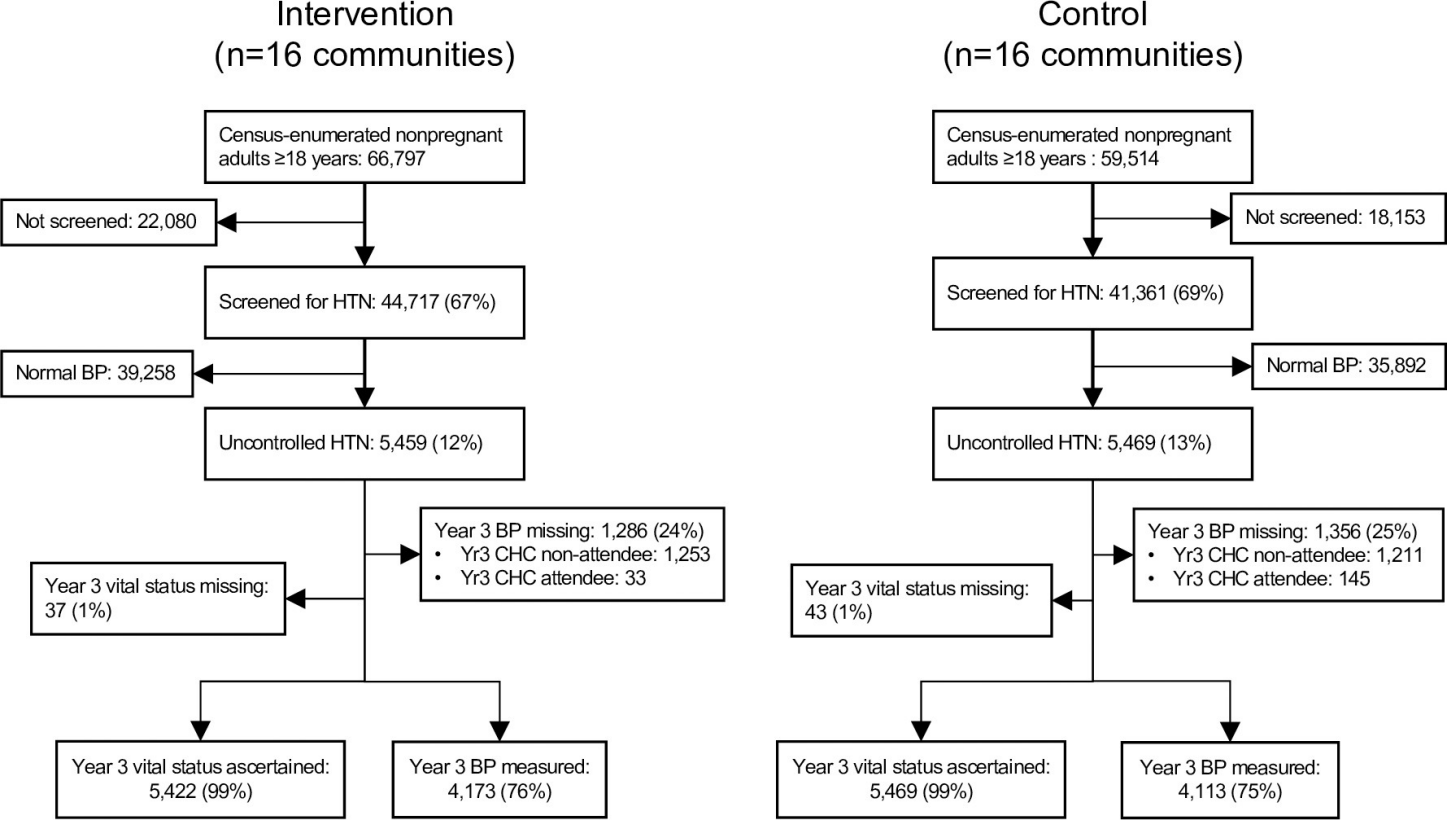
Study flow diagram. Baseline and year 3 BP was measured at CHCs. Vital status was measured in all study participants regardless of CHC participation. BP, blood pressure; CHC, community health campaign; HTN, hypertension; yr3, year 3.

We conducted baseline hypertension screening in 86,078 (68%) of 126,311 census-enumerated nonpregnant adults ≥18 years of age, 44,717 (67%) in intervention communities and 41,361 (69%) in control communities (Fig 1). Baseline characteristics of the screened adult population and those with uncontrolled hypertension were similar between intervention and control communities (Tables A and B in S1 Tables, Table 1). Of those screened, 12% in intervention communities (*n =* 5,459) and 13% in control communities (*n* = 5,469) had uncontrolled hypertension and were included in analysis.

**Table 1:** Baseline characteristics of nonpregnant adults with baseline uncontrolled hypertension.

	Intervention		Control		Overall	
	*n* = 5,459		*n =* 5,469		*n* = 10,928	
	n	%	n	%	n	%
Region						
Eastern Uganda	2,117	39%	2,136	39%	4,253	39%
Kenya	1,347	25%	1,439	26%	2,786	25%
Western Uganda	1,995	37%	1,894	35%	3,889	36%
Sex						
Female	3,037	56%	3,021	55%	6,058	55%
Male	2,422	44%	2,448	45%	4,870	45%
Age category						
18–29 years	630	12%	679	12%	1,309	12%
30–44 years	1,195	22%	1,117	20%	2,312	21%
45–59 years	1,549	28%	1,568	29%	3,117	29%
60–74 years	1,440	26%	1,448	26%	2,888	26%
≥75 years	645	12%	657	12%	1,302	12%
Wealth quintile categories*						
First, indicating least wealth	933	17%	1,033	19%	1,966	18%
Second	902	17%	982	18%	1,884	17%
Third	1,088	20%	1,086	20%	2,174	20%
Fourth	1,178	22%	1,205	22%	2,383	22%
Fifth, indicating most wealth	1,329	24%	1,111	20%	2,440	22%
Missing	29	1%	52	1%	81	1%
Self-reported prior HTN diagnosis	690	13%	770	14%	1,460	13%
Self-reported baseline HTN treatment	364	7%	413	8%	777	7%
Baseline hypertension grade^†^						
Grade 1 (140–159/90–99)	3,810	70%	3,775	69%	7,585	69%
Grade 2 (160–179/100–109)	1,197	22%	1,162	21%	2,359	22%
Grade 3 (≥180/110)	452	8%	532	10%	984	9%
Baseline BMI^‡^						
Underweight (<18.5)	836	15%	807	15%	1,643	15%
Healthy weight (18.5–24.9)	3,288	60%	3,205	59%	6,493	59%
Overweight (25.0–29.9)	946	17%	1,040	19%	1,986	18%
Obese (≥30)	368	7%	348	6%	716	7%
Missing	21	0%	69	1%	90	1%
Comorbid conditions						
HIV	357	7%	320	6%	677	6%
Diabetes	227	4%	250	5%	477	4%

*Quintiles calculated using principle component analysis of baseline household wealth survey and were calculated at the level of the household.

^†^Baseline hypertension severity defined by lowest of 3 BP measurements and classified as Grade 1 (BP 140–159/90–99 mm Hg), Grade 2 (BP 160–179/100–109 mm Hg), Grade 3 (BP ≥180/110 mm Hg).

^‡^BMI categories include underweight (BMI <18.5 kg/m^2^), normal (BMI 18.5–24.9 kg/m^2^), overweight (BMI 25–29.9 kg/m^2^), or obese (BMI ≥30 kg/m^2^).

BMI, body mass index; BP, blood pressure; HTN, hypertension.

Among 10,928 participants with baseline uncontrolled hypertension, 55% were women (*n =* 6,058) and median age was 53 years (25th to 75th percentile 40 to 66). Only 13% reported a prior diagnosis of hypertension (*n =* 1,460), 53% of whom reported current hypertension treatment (*n =* 777). The majority had grade 1 hypertension (69%, *n =* 7,585), 22% had grade 2 hypertension (*n =* 2,359), and 9% had grade 3 hypertension (*n =* 984). About 4% had comorbid diabetes (*n =* 477), and 6% had comorbid HIV (*n =* 677); among whom baseline CD4 count was <350 cells/mL in 23% (*n =* 156), 350 to 499 cells/mL in 23% (*n =* 155), ≥500 cells/mL in 52% (*n* = 355), and missing in 2% (*n* = 11). Baseline HIV viral suppression (<500 copies/mL) among those with a measured viral load was 59% (306/521).

Compared to males, females with baseline uncontrolled hypertension were older (43% age ≥60 [2,589/6,058] versus 33% for males [1,601/4,870]), had more severe hypertension (35% with grade 2 or greater [2,113/6,058] versus 25% for males [1,230/4,870]), more likely to report a prior diagnosis of hypertension (18% [1,061/6,058] versus 9% [399/4,870]), and more likely to be obese (10% [581/6,058] versus 3% [135/4,870]) (Table C in S1 Tables).

### Mortality

**Fig 2:**
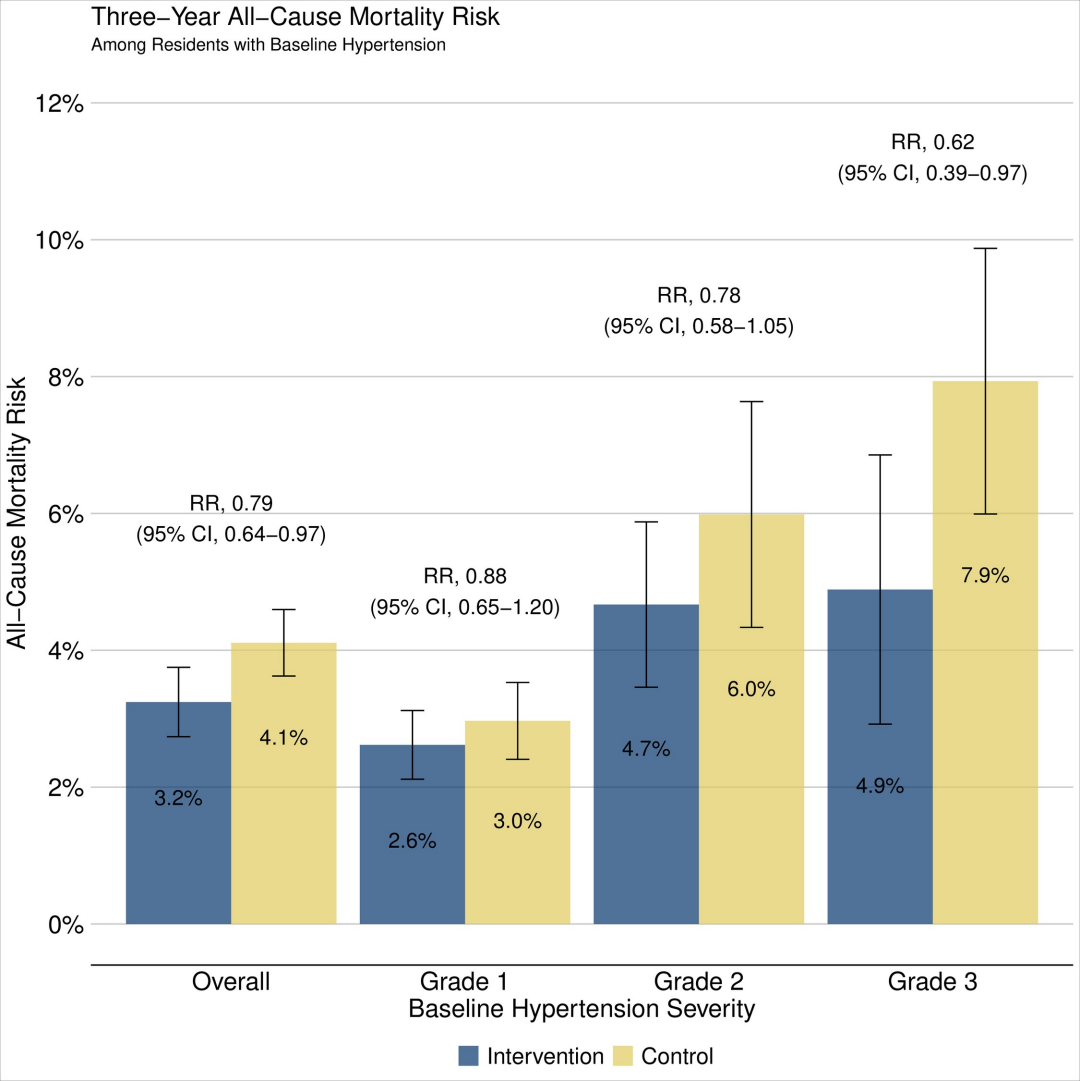
Cumulative incidence of mortality by year 3. Estimates obtained using 2-stage TMLE to estimate and compare community-level mortality by 3 years. Vertical error bars depict arm-specific 95% CIs. Baseline hypertension severity defined by lowest of 3 BP measurements and classified as Grade 1 (BP 140–159/90–99 mm Hg), Grade 2 (BP 160–179/100–109 mm Hg), Grade 3 (BP ≥180/110 mm Hg). BP, blood pressure; CI, confidence interval; RR, relative risk; TMLE, targeted maximum likelihood estimation.

**Fig 3:**
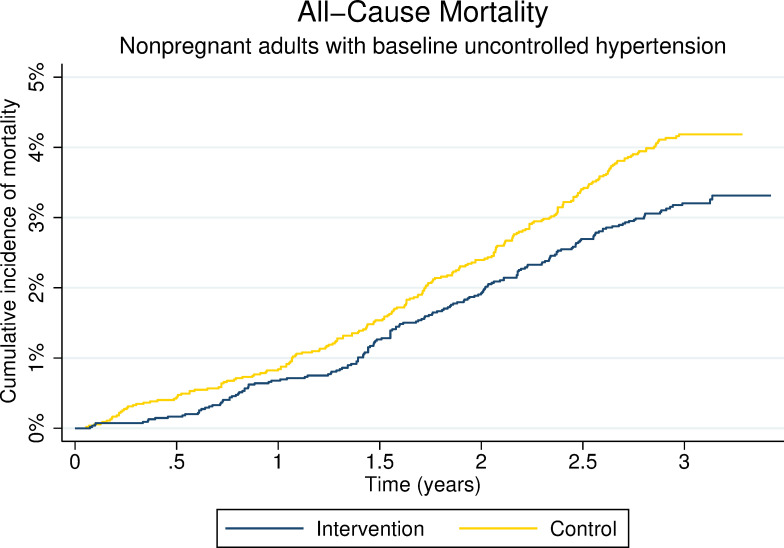
Kaplan–Meier curve depicting cumulative incidence of mortality by trial arm. Participants with unknown year 3 vital status were censored at the time they were last known to be alive.

Vital status at year 3 was ascertained in 99% of participants in both intervention communities (5,422/5,459) and control communities (5,426/5,469) (Fig 1). A total of 399 participants died over the 3 years of follow-up, 174 in intervention communities and 225 in control communities. Three-year cumulative mortality risk was 3.2% (174/5,422) in the intervention arm and 4.1% (225/5,426) in the control arm, corresponding to a 21% reduction in 3-year mortality in communities that received the SEARCH intervention (aRR 0.79, 95% confidence interval (CI) 0.64 to 0.97, *p* = 0.028) (Figs 2 and 3, Table D in S1 Tables). Most participants died of illness (164/174 [94%] in the intervention group and 218/225 [97%] in the control group; Table E in S1 Tables).

Mortality increased in a dose-dependent fashion with baseline hypertension severity, and intervention effect was greater with increasing baseline grade of hypertension (Fig 2, Table D in S1 Tables). Among those with grade 3 hypertension at baseline (≥180/110 mm Hg), 3-year mortality was 4.9% (22/451) in the intervention group and 7.9% (42/527) in the control group (aRR 0.62, 95% CI 0.39–0.97, *p* = 0.038). In sensitivity analyses, relative mortality reductions were greater when excluding adults ≥80 years of age (Fig A in S1 Figs, Table F in S1 Tables). Results were similar when excluding those with prevalent HIV infection (Fig B in S1 Figs, Table G in S1 Tables), when stratified by sex (Fig C in S1 Figs), and when stratified by country (Figure D in S1 Figs).

### Population-level hypertension control at year 3

**Fig 4:**
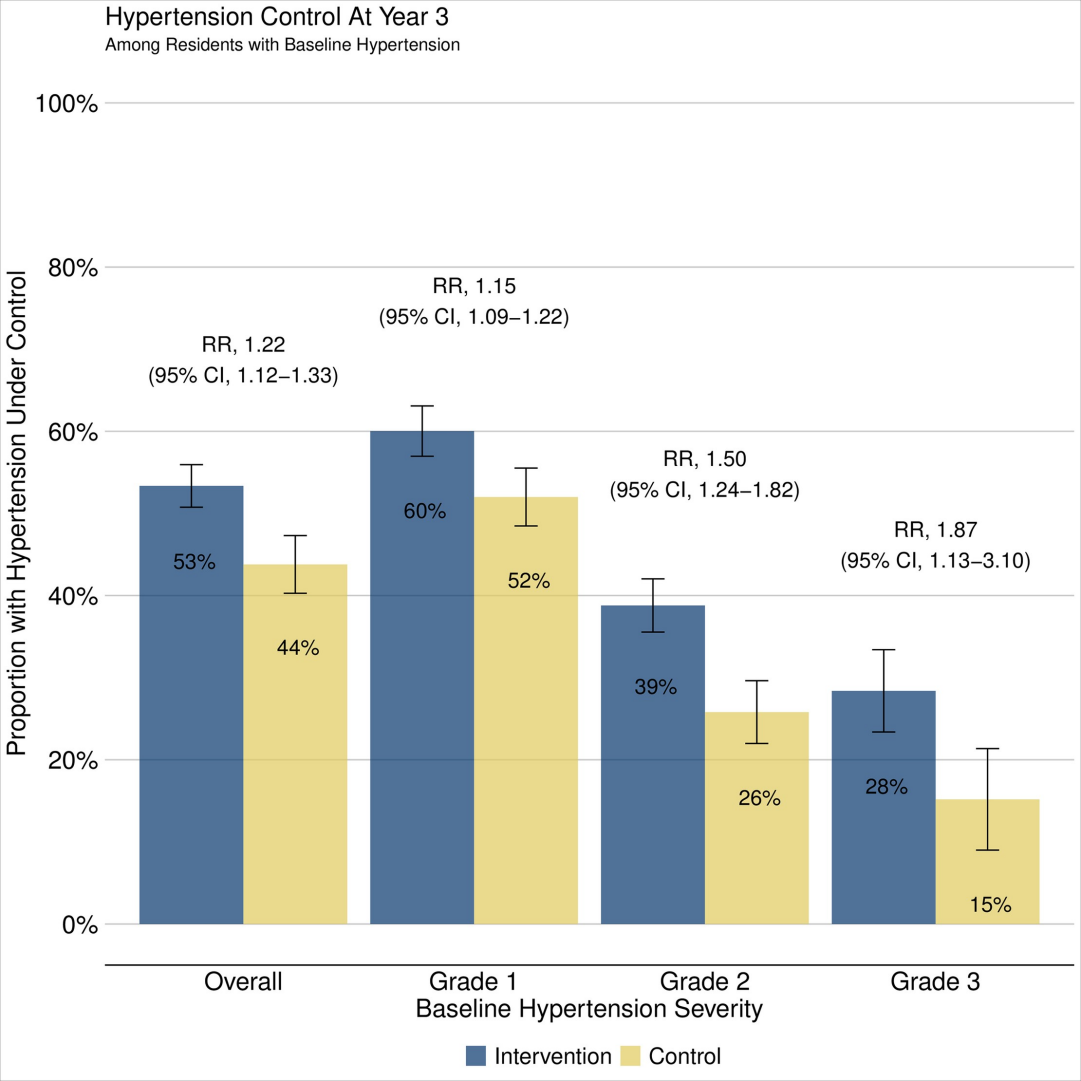
Hypertension control at year 3. Estimates obtained using 2-stage TMLE to estimate and compare community-level hypertension control at year 3 population-level BP measurement. Vertical error bars depict arm-specific 95% CIs. Hypertension control defined as lowest of 3 BPs <140/90 mm Hg at year 3 follow-up measurement. Baseline hypertension severity defined by lowest of 3 BP measurements and classified as Grade 1 (BP 140–159/90–99 mm Hg), Grade 2 (BP 160–179/100–109 mm Hg), Grade 3 (BP ≥180/110 mm Hg). BP, blood pressure; CI, confidence interval; RR, relative risk; TMLE, targeted maximum likelihood estimation.

Blood pressure was measured at year 3 community health campaigns in 76% of participants in intervention communities (4,173/5,459) and 75% in control communities (4,113/5,469); there were no meaningful differences between the participants measured and the participants missed (Table H in S1 Tables). Overall, 53% of intervention group participants and 44% of control group participants achieved hypertension control at year 3 (aRR 1.22, 95% CI 1.12 to 1.33, *p* < 0.001) (Fig 4, Table I in S1 Tables). The intervention both improved hypertension control and reduced the severity of hypertension at year 3 across all strata of baseline severity, with larger magnitudes of effect among persons with more severe baseline hypertension (Table 2, Table J in S1 Tables). Among persons with baseline grade 3 hypertension (≥180/110 mm Hg), 79% in the intervention group versus 61% in the control group had reduced hypertension severity after 3 years. The intervention effect on hypertension control was similar by sex (Fig E in S1 Figs) and when stratified by country (Fig F in S1 Figs).

**Table 2:** Change in HTN severity from baseline to year 3.

	Year 3 HTN Severity				
Baseline HTN Severity	Controlled	Grade 1	Grade 2	Grade 3	Total
**Intervention (Overall)**	**2,200 (53%)**	**1,290 (31%)**	**511 (12%)**	**172 (4%)**	**4,173**
**Grade 1**	1,752 (60%)	862 (29%)	263 (9%)	47 (2%)	2,924 (70%)
**Grade 2**	351 (39%)	329 (36%)	172 (19%)	54 (6%)	906 (22%)
**Grade 3**	97 (28%)	99 (29%)	76 (22%)	71 (21%)	343 (8%)
**Control (Overall)**	**1,764 (43%)**	**1,371 (33%)**	**639 (16%)**	**339 (8%)**	**4,113**
**Grade 1**	1,475 (52%)	987 (35%)	317 (11%)	65 (2%)	2,844 (69%)
**Grade 2**	227 (26%)	312 (35%)	218 (25%)	123 (14%)	880 (21%)
**Grade 3**	62 (16%)	72 (19%)	104 (27%)	151 (39%)	389 (9%)

HTN severity defined by lowest of 3 BP measurements and classified as Controlled (BP <140/90 mm Hg), Grade 1 (BP 140–159/90–99 mm Hg), Grade 2 (BP 160–179/100–109 mm Hg), Grade 3 (BP ≥180/110 mm Hg). Rows represent baseline HTN severity, and columns represent year 3 HTN severity; both baseline and year 3 BP measurements were conducted at community health campaigns, and, thus, measurement was independent of engagement with clinical care.

BP, blood pressure; HTN, hypertension.

### Hypertension care cascade

**Fig 5:**
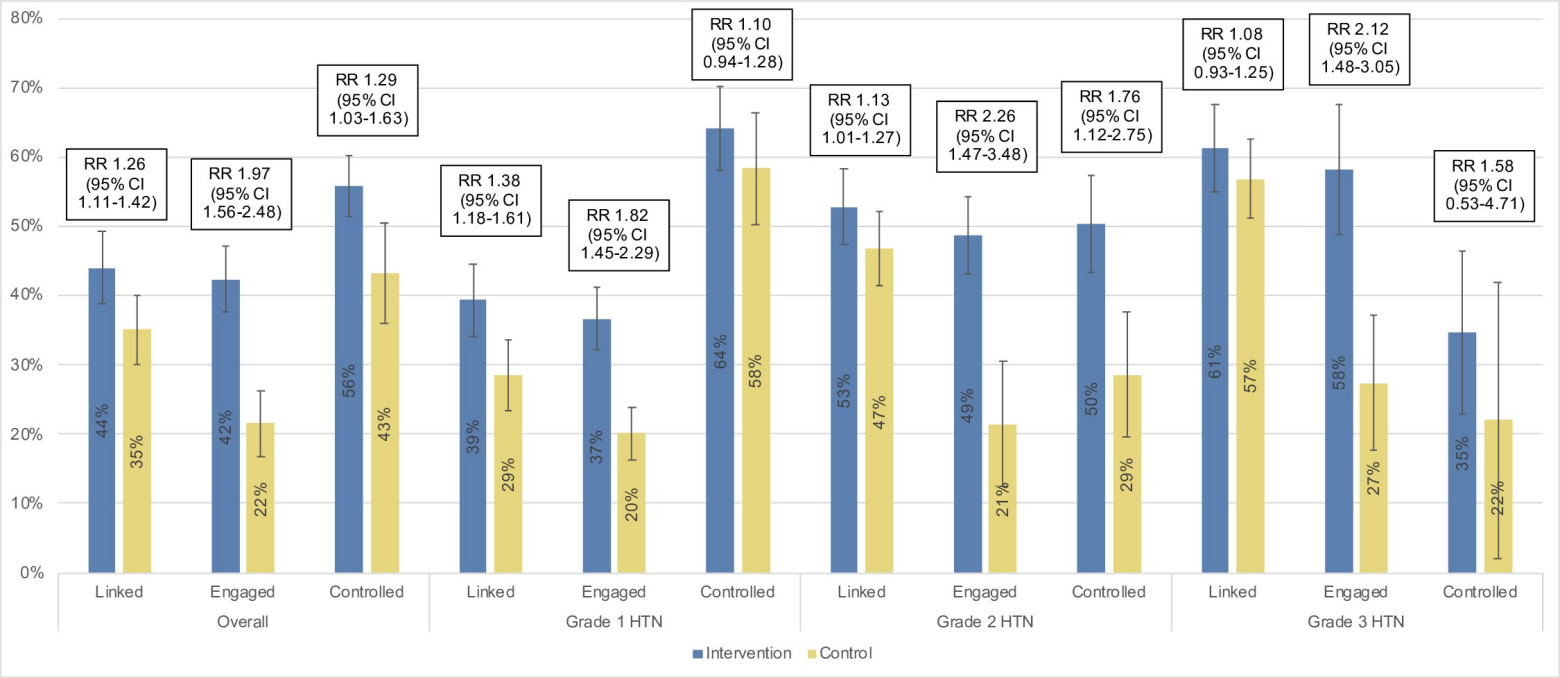
Cascade of hypertension care in 20 communities in Uganda. Figure represents the proportion attaining each cascade step, among those attaining the prior step. Estimates obtained using 2-stage TMLE to estimate and compare the community-level proportion attaining each cascade step. Vertical error bars depict arm-specific 95% CIs. Linkage to care defined as ≥1 visit for hypertension care in the first year after baseline hypertension screening. Engagement in care defined as ≥1 clinic visit for hypertension care in each of 3 years of study follow-up. Hypertension control defined as the lowest of 3 BP measurements <140/90 mm Hg at year 3 community-wide hypertension testing. Baseline hypertension severity defined by lowest of 3 baseline BP measurements and classified as Grade 1 (BP 140–159/90–99 mm Hg), Grade 2 (BP 160–179/100–109 mm Hg), and Grade 3 (BP ≥180/110 mm Hg). BP, blood pressure; CI confidence interval; HTN, hypertension; RR, relative risk; TMLE, targeted maximum likelihood estimation.

In Uganda, 44% of intervention group participants (1,810/4,112) and 35% of control group participants (1,413/4,030) linked to care within the first year following baseline screening (aRR 1.26, 95% CI 1.11 to 1.42, *p* = 0.002) (Fig 5), with similar intervention effect stratified by sex (Fig G in S1 Figs). Among those who linked to care, 42% of intervention group participants and 22% of control group participants attended at least 1 clinic visit in each of the 3 follow-up years (aRR 1.97, 95% CI 1.56 to 2.48, *p* < 0.001; Fig 5, Fig H in S1 Figs). Among those engaged in care, 56% of intervention group participants and 43% of control group participants had controlled hypertension at year 3 (aRR 1.29, 95% CI 1.03 to 1.63, *p* = 0.033). Visual examination of Fig 5 indicates that linkage and care engagement increased with greater baseline hypertension severity, while hypertension control among those engaged in care decreased with greater hypertension severity.

### Predictors of mortality, hypertension control, and linkage to care

**Fig 6:**
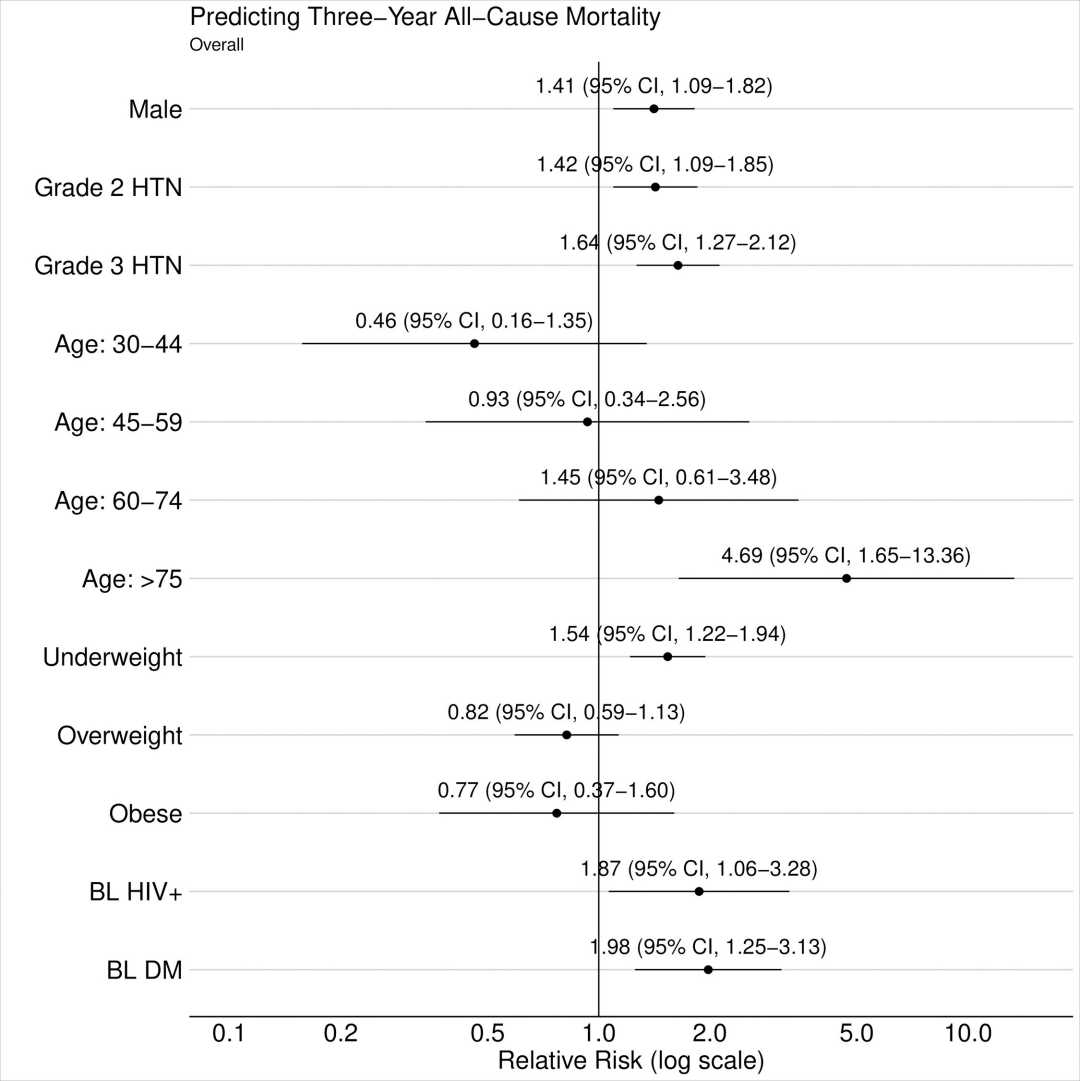
Predictors of 3-year mortality. Predictors of mortality by study year 3 using multivariable TMLE, with relative risks for each variable compared to reference value. Horizontal error bars depict predictor-specific 95% CIs. Reference values for relevant categorical variables include female, grade 1 hypertension, age 18–29 years, normal BMI, baseline HIV uninfected, and baseline absence of diabetes. Baseline hypertension severity defined by lowest of 3 BP measurements and classified as Grade 1 (BP 140–159/90–99 mm Hg), Grade 2 (BP 160–179/100–109 mm Hg), Grade 3 (BP ≥180/110 mm Hg). BMI categories include underweight (BMI <18.5 kg/m^2^), normal (BMI 18.5–24.9 kg/m^2^), overweight (BMI 25–29.9 kg/m^2^), or obese (BMI ≥30 kg/m^2^). BL, baseline; BMI, body mass index; BP, blood pressure; CI, confidence interval; DM, diabetes mellitus; HTN, hypertension; TMLE, targeted maximum likelihood estimation.

Predictors of mortality among adults with baseline hypertension included age ≥75 years (aRR 4.69, 95% CI 1.65 to 13.36, *p* = 0.005), male sex (aRR 1.41, 95% CI 1.09 to 1.82, *p* = 0.009), baseline HIV infection (aRR 1.87, 95% CI 1.06 to 3.28, *p* = 0.031), underweight (BMI <18.5 kg/m^2^; aRR 1.54, 95% CI 1.22 to 1.94, *p* < 0.001), and baseline diabetes (aRR 1.98, 95% CI 1.25 to 3.13, *p* = 0.005) (Fig 6, Table K in S1 Tables). Greater severity of baseline hypertension also increased risk for mortality, with 42% greater mortality for grade 2 (aRR 1.42, 95% CI 1.09 to 1.85, *p* = 0.010) and 64% greater mortality for grade 3 (aRR 1.64, 95% CI 1.27 to 2.12, *p* < 0.001), compared to baseline grade 1 hypertension.

Persons with greater baseline hypertension severity were at lower risk of achieving hypertension control at year 3 (aRR 0.59, 95% CI 0.55 to 0.64, *p* < 0.001 for grade 2 and aRR 0.44, 95% CI 0.35 to 0.55, *p* < 0.001 for grade 3) (Fig I in S1 Figs, Table L in S1 Tables). HIV-infected individuals were more likely to achieve hypertension control than those who were HIV uninfected at baseline (aRR 1.24, 95% CI 1.15 to 1.33, *p* < 0.001), while obese individuals were less likely to achieve hypertension control (BMI ≥30 kg/m^2^; aRR 0.84, 95% CI 0.75 to 0.95, *p* = 0.009).

Linkage to care among participants in Uganda was greater with older age (aRR for age >75 1.71, 95% CI 1.44 to 2.02, *p* < 0.001), higher baseline hypertension severity (aRR 1.34, 95% CI 1.26 to 1.43, *p* < 0.001 for grade 2 and aRR 1.63, 95% CI 1.46 to 1.83, *p* < 0.001 for grade 3), HIV infection (aRR 2.58, 95% CI 2.01 to 3.30, *p* < 0.001), and diabetes (aRR 1.34, 95% CI 1.21 to 1.49, *p* < 0.001) (Fig J in S1 Figs, Table M in S1 Tables).

## Discussion

Following community-based hypertension screening that reached 86,078 adults in 32 rural communities in Uganda and Kenya, implementation of a patient-centered, streamlined hypertension care model integrated with the existing HIV care delivery system was associated with a 21% reduction in 3-year mortality among nonpregnant adults with baseline uncontrolled hypertension, compared to optimized standard of care in control communities. Mortality reductions were greatest among those with baseline grade 3 hypertension (blood pressure ≥180/110 mm Hg), highlighting the impact over a relatively short time period of identifying individuals with severe elevations in blood pressure and linking them to a care system that actively seeks to reduce patient- and clinic-level barriers.

At study baseline, a community-based multidisease model was used to integrate hypertension and diabetes screening into community-wide HIV testing, successfully screening 68% of 126,311 census-enumerated adults for hypertension. As we previously reported, this was achieved at a marginal cost increase of $1.16 USD per person [27]. Of those identified with uncontrolled hypertension, 87% were previously unaware of their diagnosis, consistent with prior studies demonstrating very low hypertension awareness in SSA [5,6]. Although other studies have demonstrated feasibility of integrating HIV and NCD community-based screening [28,29], we demonstrate that community-based multidisease testing campaigns can achieve a high level of population-wide hypertension screening coverage, addressing the critical first step in the hypertension care cascade.

In the context of this population-wide screening, offered in both randomized arms at baseline, we found that a patient-centered chronic care delivery model improved hypertension control and reduced mortality among adults with uncontrolled hypertension beyond that of optimized standard care. We previously reported profound population-level health effects of this care delivery model among HIV-infected persons, including reduced mortality among those with prevalent HIV infection [17] and men with low CD4 counts [30], improved viral suppression among those with HIV viremia despite prior attempts at care engagement [31], reduced maternal-to-child transmission of HIV [32], and decreased incidence of tuberculosis [17]. Taken together, our findings support the value of integrated patient-centered chronic care models, when combined with multidisease community-based screening programs, for improving a wide range of population health outcomes.

Through a patient-centered approach, our intervention targeted multiple barriers along the hypertension care cascade, improving hypertension diagnosis, linkage to care, and hypertension control. The intervention included introduction to a clinic staff member at the time of hypertension diagnosis to facilitate relationship-building and promote linkage. At the clinic and provider levels, the intervention included provider training on providing friendly services and fostering a welcoming environment, clinic procedures to expedite visits and minimize patient waiting time, and multimonth medication refills. We have previously reported that HIV-infected patients identified increased support and access to providers available in our patient-centered care model as important for improving care engagement [33]; further research is needed to understand the elements of this care model that were most important for improving engagement with hypertension care. Our findings build on prior literature demonstrating that patient-centered care models can improve patient engagement in care and health outcomes [34].

Our findings help narrow the knowledge–implementation gap for translating evidence-based hypertension treatment into practice in SSA. Despite clear evidence of the efficacy of antihypertensive treatment [7,8], our study is the first, to our knowledge, to demonstrate reductions in population-level mortality through implementation of an integrated hypertension–HIV chronic care model in SSA. Other clinic-based NCD care models have shown modest improvement in clinic processes or in blood pressure control with task-shifting to nonphysician health workers in nonintegrated settings [35,36] and with integration of hypertension into HIV care [13–16]; ultimately, multiple models will likely be needed to adapt hypertension care into different contexts. Interventions that incorporate population-level community-based NCD screening have been more limited. Outside of SSA, the HOPE 4 trial recently demonstrated reduction in population-level cardiovascular risk with a community-based lay health worker–delivered hypertension intervention in Malaysia and Colombia [37]. Our study expands on these findings to demonstrate the effectiveness of a patient-centered chronic care model that leverages the HIV care system for improving population-level hypertension control and reducing mortality within the context of population-level NCD and HIV screening.

Although improvements in hypertension treatment were sufficient to reduce mortality, there are several opportunities for further improvement. First, linkage to care was suboptimal, though similar to that observed in smaller community-based hypertension screening studies [29,38]. Individuals with comorbid HIV linked at much higher rates than those without HIV, a difference that may be attributable in part to enhanced linkage interventions received by persons with HIV infection, including receipt of a transport voucher at time of diagnosis (both arms) and a phone call and/or home visit for persons who missed their scheduled intake appointment (intervention arm only). Interventions to improve linkage among people with hypertension would likely further reduce mortality beyond what we observed in the SEARCH study. Second, as previously reported, limited medication supplies intermittently resulted in shorter duration of medications dispensed than intended, contributing to reduced hypertension control [19]. Improvements in the hypertension medication supply chain, informed by success with the HIV supply chain, may further improve care engagement and hypertension control beyond what was achieved in the SEARCH study [39]. Third, treatment algorithms in SEARCH used sequential addition of single antihypertensive medications, some of which involved twice daily dosing. Use of once daily fixed-dose combination therapy for hypertension treatment may further improve hypertension control through simplified implementation, increased treatment efficacy, and improved medication adherence [40]. Finally, engagement in hypertension care declined over time. Additional research is needed to understand the determinants of disengagement from hypertension care and areas for possible intervention.

A recent cost-effectiveness analysis found that scale-up of an integrated NCD-HIV intervention similar to SEARCH would avert 116,600 CVD events and 43,600 cardiovascular deaths across Kenya over the next 15 years and would be highly cost-effective at $860 USD per disability-adjusted life year (DALY) averted [41]. However, these estimates were based on hypertension costs from nonintegrated care settings that were much higher than costs reported in SEARCH. We estimate that hypertension care cost an additional $11.39 USD/person/year for HIV-uninfected persons and $6.29 USD/person/year for HIV-infected persons [42], far lower than estimates of $77.65 USD/person/year reported from nonintegrated settings that were used in this cost-effectiveness analysis. Thus, the SEARCH universal screening and streamlined care model may be more cost-effective than previously estimated.

This study had several limitations. First, vital status was assessed among 99% of participants at the end of the study with broad causes of mortality identified using methods previously developed (e.g., illness, childbirth, suicide, accident) [43]; however, we were not able to identify more specific causes of mortality that are known to be associated with hypertension (e.g., myocardial infarction, cerebrovascular accident). Nonetheless, we observed a population-level reduction in all-cause mortality with implementation patient-centered hypertension treatment with greater impact among those with more severe baseline hypertension. Second, unavailability of visit data in Kenya precluded us from estimating linkage and care engagement across the entire study population. Assessment of these process outcomes in 20 communities in Uganda provided insight into the mechanisms involved in observed reductions in mortality and hypertension control; importantly, the core health outcomes assessed in this analysis (mortality and hypertension control) were assessed at a population level with high coverage, including among persons not in care, in all 32 study communities. Third, we were only able to measure linkage to care at clinics within study communities; thus, linkage to clinics outside of study communities may have been missed. If improvements in care quality due to the patient-centered care intervention led participants in intervention verus control communities to disproportionately link to study-supported government clinics rather than to other clinics, our results may overestimate the intervention impact on linkage. Finally, the control condition in our study exceeded the contemporary standard of hypertension care; thus, our findings likely underestimate the effects of implementing this population-level multidisease test and treat intervention. Both control and intervention communities received community-wide hypertension and multidisease screening, substantially increasing hypertension diagnosis. Intervention and control community clinics were all government run and received similar levels of staffing support provided by the study in order to ensure that hypertension care was delivered per intended country guidelines in control communities. Medications for hypertension were also provided to patients free of charge in control communities, concordant with country guidelines but exceeding conditions in usual practice where patients are usually required to purchase medications. Further research is needed to evaluate implementation strategies to translate the SEARCH patient-centered chronic care model into routine practice settings.

## Conclusions

In conclusion, we found that patient-centered hypertension treatment integrated into the HIV care delivery system improved all-cause mortality and hypertension control among adults with uncontrolled hypertension identified through community-based multidisease screening. Broader, sustained adoption of the SEARCH patient-centered chronic care model is a cost-effective way to improve NCD treatment in settings with a high burden of HIV and may result in greater reduction in CVD and mortality over time than was observed over this 3-year study. Further, improving community health through implementation of a chronic care model that integrates NCD and HIV treatment makes important strides toward universal primary healthcare access and achieving Sustainable Development Goals to reduce premature mortality from NCDs.

## Supporting information

S1 Consort Checklist Consort Statement checklist with extension for cluster designs. (DOCX)

S1 File Hypertension algorithm. Hypertension diagnosis and treatment algorithms used in the study.
(PDF)

S2 File Diabetes algorithm. Diabetes diagnosis and treatment algorithms used in the study.
(PDF)

S1 Statistical Analysis Plan (PDF)

S1 Tables Supporting tables. Table A. Characteristics of population screened for hypertension at study baseline. Table B. Risk factors for baseline uncontrolled hypertension among overall screened population. Table C. Baseline characteristics, stratified by sex. Table D. Mortality by year 3 among all adults with baseline uncontrolled hypertension. Table E. Causes of death by trial arm. Table F. Mortality by year 3 among adults <80 years of age with baseline uncontrolled hypertension. Table G. Mortality by year 3 among HIV-uninfected adults with baseline uncontrolled hypertension. Table H. Characteristics of participants with measured year 3 blood pressure. Table I. Year 3 hypertension control, by baseline hypertension severity. Table J. Change in blood pressure from baseline to study year 3. Table K. Baseline characteristics of participants who died by study year 3. Table L. Baseline characteristics of participants with controlled hypertension at study year 3. Table M. Baseline characteristics of participants who linked to care within 1 year of baseline hypertension screening (Uganda only).
(PDF)

S1 Figs Supporting figures. Fig A. Mortality by year 3, among adults <80 years of age. Fig B. Mortality by year 3, among HIV-uninfected adults. Fig C. Mortality by year 3, stratified by sex. Fig D. Mortality by year 3, stratified by country. Fig E. Year 3 hypertension control, stratified by sex. Fig F. Year 3 hypertension control, stratified by country. Fig G. Linkage to hypertension care in Uganda, stratified by sex. Fig H. Care engagement among those linked to care in Uganda. Fig I. Predictors of year 3 hypertension control. Fig J. Predictors of linkage to care (Uganda only).
(PDF)

## Abbreviations

aRR: adjusted relative risk
BMI: body mass index
CI: confidence interval
CVD: cardiovascular disease
DALY: disability-adjusted life year
NCD: noncommunicable disease
SEARCH: Sustainable East Africa Research in Community Health
SSA: sub-Saharan Africa
TMLE: targeted maximum likelihood estimation
