# Supplementary material for: Effect of a patient-centered hypertension delivery strategy on all-cause mortality: Secondary analysis of SEARCH, a community-randomized trial in rural Kenya and Uganda
Source: PLoS Med. 2021 Sep 20;18(9):e1003803. doi: 10.1371/journal.pmed.1003803 (PMC8489716; doi:10.1371/journal.pmed.1003803)
Supplement: S1 File — Hypertension diagnosis and treatment algorithms used in the study. (PDF) [file pmed.1003803.s002.pdf]

SEARCH Hypertension Diagnosis and Treatment Algorithms

Hypertension Diagnosis Algorithm

Measure **BP** in **ALL** adults

SBP >140 or DBP >90

Check again 3 times at 1 minute intervals

If any screening BP has SBP<140 and DBP<90 → Evaluate again after 6 month

High BP after 3 screenings

Ask about symptoms

Shortness of breath on small exercises, feet swollen, chest Pain, limb weakness/paralysis, blurred vision, headache or convulsions.

If "Yes" to any symptoms → Call ART Coordinator or Refer patient to hospital

No symptoms

Check cardiovascular risk factors

a) Proteinuria (on dipstick)

b) Diabetic (RBS>11.1mmoles)

If "Positive" on either test → Call ART Coordinator or Refer patient to hospital

No risk factors

SBP 140-159 and/or DBP 90-99	SBP 160-179 and/or DBP 100-109	SBP >180 or DBP > 110
3-month trial of **lifestyle changes**:<br><br>Weight reduction, increase physical exercise, decrease dietary salt intake, decrease alcohol consumption, stop smoking promote fresh fruits and vegetables intake, fatty food limitation<br><br>**Follow up in 3 months** - If BP remains high then start medications	**Lifestyle changes**:<br>Weight reduction, increase physical exercise, decrease dietary salt intake, decrease alcohol consumption, stop smoking, promote fresh fruits and vegetables intake, fatty food limitation<br><br>**PLUS**<br><br>**Medications**<br>(Follow treatment algorithm)	Call ART Coordinator<br>or<br>Refer patient to hospital

Hypertension Treatment Algorithm

Initial medication regimen

Bendroflumethiazide 5mg daily

Follow-up in **4 weeks**

↓

Target BP not achieved?

Increase
Bendroflumethiazide 10mg
Follow-up in **4 weeks**

↓

Target BP not achieved?

Bendroflumethiazide 10 mg + Nifedipine 20 mg BID

Follow-up in **4 weeks**

↓

Target BP not achieved?

Bendroflumethiazide 10 mg + Nifedipine 20 mg BID +
Add **Captopril 25 mg BID**

Follow-up in **4 weeks**

↓

Call ART Coordinator
or
Refer patient to the hospital

BP GOALS

Age <60 or TOD: SBP <140 and DBP <90

Age ≥60: SBP <150 and DBP<90

TARGET BP ACHIEVED?

Prescribe same treatment and follow up every 3 months

Measure BP & weight at each visit

Medication Adherence:
Always check for drug adherence **at each visit.** Ask whether patient has **taken medication on each of the last three days**. Provide **counseling** if patient has **missed any dose** in the last three days.

Lifestyle and Behavior
Review life style and Behavior. Provide Counseling if still present

Medication Tolerability:
If patient is having **side effects**, change to another medication. Alternatively, call ART coordinator or refer patient to hospital

Key
BID-Twice a day; SB -Systolic; DBP-Diastolic BP; TOD-target organ damage (Heart failure, kidney failure, stroke, myocardial infarction etc)
