# Supplementary material for: Effect of a patient-centered hypertension delivery strategy on all-cause mortality: Secondary analysis of SEARCH, a community-randomized trial in rural Kenya and Uganda
Source: PLoS Med. 2021 Sep 20;18(9):e1003803. doi: 10.1371/journal.pmed.1003803 (PMC8489716; doi:10.1371/journal.pmed.1003803)
Supplement: S2 File — Diabetes diagnosis and treatment algorithms used in the study. (PDF) [file pmed.1003803.s003.pdf]

Diabetes Diagnosis Algorithm

Measure random blood sugar (RBS) or fasting blood sugar (FBS) in adults with:

- Hypertension
- Overweight/Obesity
- Family history of diabetes
- Frequent urination
- Unusual thirst
- Weight loss
- Fatigue
- Frequent infections
- Blurred vision
- Poor wound healing
- High alcohol consumption

RBS >11 or FBS >7

Patient has Diabetes

RBS >22 → Call ART Coordinator or Refer patient to hospital

RBS 11-22 ↓

Counseling

- Weight loss
- Low calorie diet
- Increase aerobic exercise
- Stop smoking
- Decrease alcohol consumption
- Education on complications such as neuropathy, ophthalmopathy, infections
- Drug adherence education
- Glucose monitoring

Treatment

Lifestyle changes
(per counseling)

+

Medication
(follow treatment algorithm)

Diabetes Treatment Algorithm

Initial medication regimen

Metformin 500 mg daily

Follow-up in **2 weeks**

↓

RBS < 8 not achieved?

Metformin 500 mg BID

Follow-up in **2 weeks**

↓

RBS < 8 not achieved?

Metformin 500 mg TID

Follow-up in **2 weeks**

↓

RBS < 8 not achieved?

Call ART Coordinator
or
Refer patient to the hospital

Target Random Blood Sugar (RBS)

RBS < 8mmol/L

TARGET RBS < 8 ACHIEVED?

Prescribe same treatment and followup every 3 months

Measure RBS, BP & weight at each visit

Medication Adherence:
Always check for drug adherence **at each visit.** Ask whether patient has **taken medication on each of the last three days**. Provide **counseling** if patient has **missed any dose** in the last three days.

Medication Tolerability:
If patient is having **side effects**, call ART coordinator or refer patient to hospital
