# Supplementary material for: Effect of a patient-centered hypertension delivery strategy on all-cause mortality: Secondary analysis of SEARCH, a community-randomized trial in rural Kenya and Uganda
Source: PLoS Med. 2021 Sep 20;18(9):e1003803. doi: 10.1371/journal.pmed.1003803 (PMC8489716; doi:10.1371/journal.pmed.1003803)
Supplement: S1 Statistical Analysis Plan — (PDF) [file pmed.1003803.s004.pdf]

Sustainable East Africa Research in Community Health

Statistical Analysis Plan

Project: Mortality and blood pressure among adults with baseline uncontrolled hypertension in SEARCH

Nicholas Sim, PhD*
Matthew D. Hickey, MD**
Diane V. Havlir, MD**
Maya L. Petersen, PhD*
Laura B. Balzer, PhD***

for the SEARCH Collaboration

September 30, 2020

Version 1.0

Table of Contents

1. Overview ... 2

2.1 Evaluate the effect on three-year, all-cause mortality risk ... 2

2.2 Evaluate the effect on HTN control at year 3 ... 3

2.3 Evaluate the effect on hypertension care cascade (Uganda only) ... 4

2.4 Assess individual-level predictors of mortality, HTN control, and linkage ... 5

*School of Public Health, University of California Berkeley, Berkeley, USA
**Division of HIV, ID, & Global Medicine, Department of Medicine, UCSF, San Francisco, USA
***School of Public Health & Health Sciences, University of Massachusetts Amherst, Amherst, USA

1. Overview

SEARCH was a universal test-and-treat, cluster randomized trial, conducted in 32 rural communities Kenya and Uganda from 2013-2017.[1] In both intervention communities (n=16) and control communities (n=16), baseline multi-disease testing was conducted through a population-based, out-of-facility approach.[2] All individuals diagnosed with hypertension (HTN) were given a clinic appointment at the nearest government-run clinic; those also living with HIV were given a one-time transportation voucher. In intervention communities, participants were also introduced to clinic staff and were tracked if living with HIV and missed their first appointment. In intervention communities, HTN care was integrated into HIV care in a ‘streamlined’ model, detailed elsewhere.[3,4] In control communities, HTN care was delivered as isolated, non-integrated care. Both intervention and control communities used HTN treatment guidelines adapted from standard country guidelines.

The primary objective of this project is to determine whether the SEARCH intervention improved the three-year, all-cause mortality (henceforth referred to as “mortality") risk. Secondary objectives include evaluating the intervention effect on HTN control at year 3 (henceforth referred to as “HTN control”) and on linkage to care within one year (henceforth referred to as “linkage”). Additionally, we aim to characterize the HTN care cascade and changes in HTN severity. Finally, we evaluate predictors of mortality, HTN control, and linkage.

Throughout, the population of interest comprises non-pregnant adults, aged 18 years and older, who have uncontrolled HTN at baseline identified through population-level screening (defined as systolic blood pressure ≥140 mmHg or diastolic blood pressure ≥90 mmHg on all baseline measures). We will provide descriptions of participant flow through the study (i.e. a consort diagram), measurement coverage, and baseline characteristics (e.g. region, gender, age, socioeconomic status, prior HTN diagnosis, prior HTN treatment, HTN staging, body mass index (BMI), and comorbidities), overall and by arm and gender. We will also report risk factors (N and %) for uncontrolled HTN at baseline, overall and by gender.

2.1 Evaluate the effect on three-year, all-cause mortality risk

To estimate the intervention effect on mortality risk, we will use a two-stage approach, appropriate for cluster randomized trials,[1,5,6] where in Stage I, we estimate the mortality risk in each community, and in Stage II, we use a cluster-level TMLE (targeted maximum likelihood estimator) to estimate the intervention effect by comparing the estimated community-specific mortality risks between randomized arms, while accounting for a pair-matched design. The algorithm is detailed in Balzer et al (2018).[6]

Specifically, in Stage I, the mortality risk for each community will be estimated using the empirical proportion of adults with baseline uncontrolled HTN who have died of any cause by the third year. We will include persons identified as out-migrating from the study communities, and exclude persons whose year 3 vital status is unknown.

Next, in Stage II, we evaluate the intervention effect by using cluster-level TMLE, which provides an unbiased and more precise estimate of the intervention effect.[8] Individuals (versus communities) will be weighted equally, and we will use sample-splitting to select the optimal adjustment set from the following pre-specified set: (i) the proportion of each community with age ≥60, (ii) the proportion of each community with grade 2 or greater baseline HTN, or (iii) nothing (unadjusted). We will formally test the null hypothesis that the SEARCH intervention did not change the mortality risk (relative risk=1).

These analyses will be repeated within subgroups defined by baseline HTN severity: grade 1 (140-159/90-99 mmHg), grade 2 (160-179/100-109 mmHg), or grade 3 (≥180/110 mmHg), as well as stratified by gender. Sensitivity analyses will exclude persons aged 80 years and above, and separately, persons with HIV at baseline.

We will also provide descriptive statistics of participants who died. Finally, we will calculate and report the arm-specific mortality rates. Person-time at risk begins at baseline community-wide hypertension screening and ends at the earliest of death, or year three community-wide blood pressure measurement.

2.2 Evaluate the effect on HTN control at year 3

The same two-stage approach will be used to assess the intervention effect on HTN control (lowest blood pressure measured <140/90 mmHg) at year 3. As with baseline, we will use blood pressure measures obtained from our population-based, out-of-facility approach.[2]

In Stage I, we will estimate the community-specific proportion of adults with uncontrolled HTN at baseline, who have controlled HTN at year 3. We will use TMLE with Super Learner[7] to adjust for differences in characteristics (e.g. gender, age, BMI) between persons with measurements and persons with missing measurements (including due to out-migration).

In Stage II, estimates of control will be compared between arms with a cluster-level TMLE, weighting individuals equally and adaptively selecting the adjustment set from (i) baseline measurement coverage, (ii) baseline prevalence of uncontrolled HTN, or (iii) nothing (unadjusted). We will formally test the null hypothesis that the SEARCH

intervention did not change the proportion of our population of interest who achieved HTN control at year 3 (relative risk=1).

These analyses will be repeated within subgroups defined by baseline HTN severity, as well as stratified by gender. As before, sensitivity analyses exclude persons aged 80 and above, and separately, persons living with HIV at baseline. Additional sensitivity analysis will be conducted defining hypertension control as <140/90 mmHg among adults ≤60 years of age and <150/90 among adults >60 years of age to reflect contemporary treatment guidelines.

Additionally, we will report (N and %) changes in grade of hypertension severity and the median (with IQR) reduction in systolic blood pressure between baseline and year three between arms, overall and by hypertension severity.

2.3 Evaluate the effect on hypertension care cascade (Uganda only)
The same two-stage approach will be used to assess the intervention effect on the hypertension care cascade (linkage, engagement, control) among 20 communities (n=10 intervention and n=10 control) in Uganda. Kenyan communities are excluded because of limitations on clinic visit data.

In Stage I, we will estimate the community-specific proportion of adults with uncontrolled HTN at baseline, who link to care using the empirical proportion of adults with baseline uncontrolled HTN who linked to care (with ≥1 clinic visit within 1 year of baseline testing). We will include persons identified as out-migrating from the study communities.

In Stage II, estimates of linkage will be compared between arms with a cluster-level TMLE, weighting individuals equally and adaptively selecting the adjustment set from (i) baseline prevalence of hypertension, (ii) the proportion of each community with Grade 2 or greater baseline HTN, or (iii) nothing (unadjusted). We formally test the null hypothesis that the SEARCH intervention did change the proportion of our population of interest who linked to care (relative risk=1).

These analyses will be repeated within subgroups defined by baseline HTN severity, as well as stratified by gender. As before, sensitivity analyses exclude persons aged 80 and above, and separately, persons living with HIV at baseline.

Similarly, we will also estimate the intervention effect on (i) engagement in care (defined as at least one clinic visit for hypertension during each of the three study follow-up years) among participants who linked to care, and (ii) HTN control among participants who were engaged and linked to care.

2.4 Assess individual-level predictors of mortality, HTN control, and linkage

We will evaluate the following baseline predictors of mortality within 3 years for our population of interest: gender, HTN severity (grade), age, BMI, baseline HIV status, and baseline diabetes status. In the primary analysis, individual-level TMLE is used to obtain variable importance measures, capturing the amount of information that a given predictor provides after adjusting for the other predictors. For each of the predictors, we will report the adjusted variables importance measures on the relative scale, treating each baseline predictor in turn as the intervention variable, and the rest as the adjustment set while accounting for the region as well. In secondary analyses, we will calculate and report unadjusted (i.e. univariate) associations. All analyses account for clustering by community. We will conduct these analyses overall and stratified by randomized arm.

Analogous methods are used to evaluate the predictors of HTN control and linkage to care. Analyses of linkage restrict to Uganda only.

References

1. Havlir DV, Balzer LB, Charlebois ED, et al. HIV Testing and Treatment with the Use of a Community Health Approach in Rural Africa. *New England Journal of Medicine*. 2019;381(3):219-229.

2. Chamie G, Clark TD, Kabami J, Kadede K, Ssemmondo E, others. A hybrid mobile HIV testing approach for population-wide HIV testing in rural East Africa. *Lancet HIV*. 2016;3(3):e111-119.

3. Kwarisiima D, Atukunda M, Owaraganise A, et al. Hypertension control in integrated HIV and chronic disease clinics in Uganda in the SEARCH study. *BMC Public Health*. 2019;19(1):511. doi:10.1186/s12889-019-6838-6

4. Kwarisiima D, Kamya M, Owaraganise A, et al. High rates of viral suppression in adults and children with high CD4+ counts using a streamlined ART delivery model in the SEARCH trial in rural Uganda and Kenya. *J Int AIDS Soc*. 2017;Jul 21(20).

5. Hayes RJ, Moulton LH. *Cluster Randomised Trials*. Chapman & Hall/CRC; 2009.

6. Balzer LB, Havlir DV, Schwab J, van der Laan MJ, Petersen ML, the SEARCH Collaboration. *Statistical Analysis Plan for SEARCH Phase I: Health Outcomes among Adults*. arXiv; 2018.

7. van der Laan MJ, Polley EC, Hubbard AE. Super learner. *Stat Appl Genet Mol Biol*. 2007;6:Article25. doi:10.2202/1544-6115.1309

8. Balzer L, van der Laan MJ, Petersen M, SEARCH Collaboration. Adaptive Pre-specification in Randomized Trials With and Without Pair-Matching. *Statistics in Medicine*. 2016;35(10):4528-4545. doi:10.1002/sim.7023
