# Supplementary material for: Effect of a patient-centered hypertension delivery strategy on all-cause mortality: Secondary analysis of SEARCH, a community-randomized trial in rural Kenya and Uganda
Source: PLoS Med. 2021 Sep 20;18(9):e1003803. doi: 10.1371/journal.pmed.1003803 (PMC8489716; doi:10.1371/journal.pmed.1003803)
Supplement: S1 Tables — Table A. Characteristics of population screened for hypertension at study baseline. Table B. Risk factors for baseline uncontrolled hypertension among overall screened population. Table C. Baseline characteristics, stratified by sex. Table D. Mortality by year 3 among all adults with baseline uncontrolled hypertension. Table E. Causes of death by trial arm. Table F. Mortality by year 3 among adults <80 years of age with baseline uncontrolled hypertension. Table G. Mortality by year 3 among HIV-uninfected adults with baseline uncontrolled hypertension. Table H. Characteristics of participants with measured year 3 blood pressure. Table I. Year 3 hypertension control, by baseline hypertension severity. Table J. Change in blood pressure from baseline to study year 3. Table K. Baseline characteristics of participants who died by study year 3. Table L. Baseline characteristics of participants with controlled hypertension at study year 3. Table M. Baseline characteristics of participants who linked to care within 1 year of baseline hypertension screening (Uganda only). (PDF) [file pmed.1003803.s005.pdf]

S1 Tables. Supplemental Tables

Table A. Characteristics of population screened for hypertension at study baseline 2

Table B. Risk factors for baseline uncontrolled hypertension among overall screened population 3

Table C. Baseline characteristics, stratified by sex 4

Table D. Mortality by year three among all adults with baseline uncontrolled hypertension 5

Table E. Causes of death by trial arm 6

Table F. Mortality by year three among adults <80 years of age with baseline uncontrolled hypertension 7

Table G. Mortality by year three among HIV-uninfected adults with baseline uncontrolled hypertension 8

Table H. Characteristics of participants with measured year 3 blood pressure 9

Table I. Year three hypertension control, by baseline hypertension severity 10

Table J. Change in blood pressure from baseline to study year three 11

Table K. Baseline characteristics of participants who died by study year three 12

Table L. Baseline characteristics of participants with controlled hypertension at study year three 13

Table M. Baseline characteristics of participants who linked to care within one year of baseline hypertension screening (Uganda only) 14

Table A. Characteristics of population screened for hypertension at study baseline

	Intervention n=44,717		Control n=41,361		Overall n=86,078	
	n	%	n	%	n	%
Region						
Eastern Uganda	15,134	34%	13,693	33%	28,827	33%
Kenya	13,880	31%	13,291	32%	27,171	32%
Western Uganda	15,703	35%	14,377	35%	30,080	35%
Sex						
Female	26,029	58%	24,146	58%	50,175	58%
Male	18,688	42%	17,215	42%	35,903	42%
Age category						
18-29 yrs	16,482	37%	15,108	37%	31,590	37%
30-44 yrs	13,782	31%	12,867	31%	26,649	31%
45-59 yrs	8,290	19%	7,709	19%	15,999	19%
60-74 yrs	4,552	10%	4,195	10%	8,747	10%
≥75 yrs	1,611	4%	1,482	4%	3,093	4%
Wealth quintile categories*						
First, indicating least wealth	7,292	16%	7,468	18%	14,760	17%
Second	7,916	18%	7,632	18%	15,548	18%
Third	8,979	20%	8,598	21%	17,577	20%
Fourth	9,555	21%	8,949	22%	18,504	21%
Fifth, indicating most wealth	10,824	24%	8,460	20%	19,284	22%
Missing	151	0%	254	1%	405	0%
Self-reported prior HTN diagnosis	1,999	4%	2104	5%	4,103	5%
Self-reported baseline HTN treatment	758	2%	868	2%	1,626	2%
Baseline hypertension stage†						
Not hypertensive (<140/90)	39,258	88%	35,892	87%	75,150	87%
Grade 1 (140-159 / 90-99)	3,810	9%	3,775	9%	7,585	9%
Grade 2 (160-179 / 100-109)	1,197	3%	1,162	3%	2,359	3%
Grade 3 (≥180/110)	452	1%	532	1%	984	1%
Baseline BMI‡						
Underweight (<18.5)	7,066	16%	6,167	15%	13,233	15%
Healthy weight (18.5-24.9)	30,628	68%	28,165	68%	58,793	68%
Overweight (25.0-29.9)	5,473	12%	5,465	13%	10,938	13%
Obese (≥30)	1,350	3%	1,271	3%	2,621	3%
missing	200	0%	293	1%	493	1%
Comorbid conditions						
HIV	4,886	11%	4,313	10%	9,199	11%
Diabetes	824	2%	944	2%	1,768	2%

*Quintiles calculated using principle-component analysis of the household wealth survey and were calculated at the level of the household. †Baseline hypertension defined by lowest of three blood pressure (BP) measurements and classified as not hypertensive (<140/90 mmHg), Grade 1 (BP 140-159/90-99 mmHg), Grade 2 (BP 160-179/100-109 mmHg), Grade 3 (BP ≥180/110 mmHg). ‡Body mass index (BMI) categories include underweight (BMI <18.5 kg/m^2), normal (BMI 18.5-24.9 kg/m^2), overweight (BMI 25-29.9 kg/m^2), or obese (BMI ≥30 kg/m^2).

Table B. Risk factors for baseline uncontrolled hypertension among overall screened population

	Total Screened	Total Uncontrolled Hypertension	% Uncontrolled Hypertension
Overall	86,078	10,928	13%
Region			
Eastern Uganda	28,827	4,253	15%
Kenya	27,171	2,786	10%
Western Uganda	30,080	3,889	13%
Sex			
Female	50,175	6,058	12%
Male	35,903	4,870	14%
Age category			
18-29 yrs	31,590	1,309	4%
30-44 yrs	26,649	2,312	9%
45-59 yrs	15,999	3,117	19%
60-74 yrs	8,747	2,888	33%
≥75 yrs	3,093	1,302	42%
Wealth quintile categories*			
First, indicating least wealth	14,760	1,966	13%
Second	15,548	1,884	12%
Third	17,577	2,174	12%
Fourth	18,504	2,383	13%
Fifth, indicating most wealth	19,284	2,440	13%
Missing	405	81	20%
Self-reported prior HTN diagnosis	4,103	1,460	36%
Self-reported baseline HTN treatment	1,626	777	48%
Baseline BMI category†			
Underweight	13,233	1,643	12%
Healthy weight	58,793	6,493	11%
Overweight	10,938	1,986	18%
Obese	2,621	716	27%
missing	493	90	18%
Comorbid conditions			
HIV	9,199	677	7%
Diabetes	1,768	477	27%

*Quintiles calculated using principle-component analysis of the household wealth survey and were calculated at the level of the household. †Body mass index (BMI) categories include underweight (BMI <18.5 kg/m^2), normal (BMI 18.5-24.9 kg/m^2), overweight (BMI 25-29.9 kg/m^2), or obese (BMI ≥30 kg/m^2).

Table C. Baseline characteristics, stratified by sex

	Screened population				Baseline Uncontrolled HTN			
	Female n=50,175		Male n=35,903		Female n=6,058		Male n=4,870	
	n	Column %	n	Column %	n	Column %	n	Column %
Region								
Eastern Uganda	15,932	32%	12,895	36%	2,257	37%	1,996	41%
Kenya	16,995	34%	10,176	28%	1,738	29%	1,048	22%
Western Uganda	17,248	34%	12,832	36%	2,063	34%	1,826	37%
Age category								
18-29 yrs	17,922	36%	13,668	38%	415	7%	894	18%
30-44 yrs	15,717	31%	10,932	30%	1,160	19%	1,152	24%
45-59 yrs	9,596	19%	6,403	18%	1,894	31%	1,223	25%
60-74 yrs	5,194	10%	3,553	10%	1,800	30%	1,088	22%
≥75 yrs	1,746	3%	1,347	4%	789	13%	513	11%
Wealth quintile categories*								
First, indicating least wealth	8,825	18%	5,935	17%	1,165	19%	801	16%
Second	9,090	18%	6,458	18%	1,053	17%	831	17%
Third	10,327	21%	7,250	20%	1,198	20%	976	20%
Fourth	10,780	21%	7,724	22%	1,319	22%	1,064	22%
Fifth, indicating most wealth	10,931	22%	8,353	23%	1,283	21%	1,157	24%
Missing	222	0%	183	1%	40	1%	41	1%
Self-reported prior HTN diagnosis	3,095	6%	1008	3%	1,061	18%	399	8%
Self-reported baseline HTN treatment	1,215	2%	411	1%	558	9%	219	4%
Baseline hypertension severity†								
Not hypertensive (<140/90 mmHg)	44,117	88%	31,033	86%	-		-	
Grade 1 (140-159 / 90-99 mmHg)	3,945	8%	3,640	10%	3,945	65%	3,640	75%
Grade 2 (160-179 / 100-109 mmHg)	1,456	3%	903	3%	1,456	24%	903	19%
Grade 3 (≥180/110 mmHg)	657	1%	327	1%	657	11%	327	7%
Baseline BMI category‡								
Underweight	6,456	13%	6,777	19%	793	13%	850	17%
Healthy weight	32,859	65%	25,934	72%	3,289	54%	3,204	66%
Overweight	8,271	16%	2,667	7%	1,339	22%	647	13%
Obese	2,279	5%	342	1%	581	10%	135	3%
missing	310	1%	183	1%	56	1%	34	1%
Comorbid conditions								
HIV	6,198	12%	3,001	8%	403	7%	274	6%
Diabetes	1,023	2%	745	2%	263	4%	214	4%

Characteristics of both screened population and nonpregnant adults with baseline uncontrolled hypertension, stratified by sex. *Quintiles calculated using principle-component analysis of the household wealth survey and were calculated at the level of the household. †Baseline hypertension severity defined by lowest of three blood pressure (BP) measurements and classified as not hypertensive (<140/90 mmHg), Grade 1 (BP 140-159/90-99 mmHg), Grade 2 (BP 160-179/100-109 mmHg), Grade 3 (BP ≥180/110 mmHg). ‡Body mass index (BMI) categories include underweight (BMI <18.5 kg/m^2), normal (BMI 18.5-24.9 kg/m^2), overweight (BMI 25-29.9 kg/m^2), or obese (BMI ≥30 kg/m^2).

Table D. Mortality by year three among all adults with baseline uncontrolled hypertension

	Intervention		Control		Effect Estimate (calculated at cluster level using TMLE, see methods)
	n	%	n	%	
Vital status assessed	5422/5459	99%	5426/5469	99%	
Overall study popuation	174/5422	3.2%	225/5426	4.1%	RR 0.79, 95% CI 0.64-0.97, p=0.028
Grade 1 hypertension	98/3783	2.6%	112/3742	3.0%	RR 0.88, 95% CI 0.65-1.20, p=0.40
Grade 2 hypertension	54/1188	4.5%	71/1157	6.1%	RR=0.78, 95% CI 0.58-1.05, p=0.091
Grade 3 hypertension	22/451	4.9%	42/527	8.0%	RR=0.62, 95% CI 0.39-0.97, p=0.038

Tabulation of vital status assessment and mortality among individual participants. Baseline hypertension severity defined by lowest of three blood pressure (BP) measurements and classified as not hypertensive (<140/90 mmHg), Grade 1 (BP 140-159/90-99 mmHg), Grade 2 (BP 160-179/100-109 mmHg), Grade 3 (BP ≥180/110 mmHg). TMLE, targeted maximum likelihood estimation.

Table E. Causes of death by trial arm

Cause of death	Intervention	Control
Illness	164 (94%)	218 (97%)
Accident	10 (6%)	1 (0%)
Homicide	0 (0%)	2 (1%)
Suicide	0 (0%)	2 (1%)
Childbirth	0 (0%)	1 (0%)
Unknown	0 (0%)	1 (0%)
Total	174	225

Table F. Mortality by year three among adults <80 years of age with baseline uncontrolled hypertension

	Intervention		Control		Effect Estimate (calculated at cluster level using TMLE, see methods)
	n	%	n	%	
Vital status assessed	5068/5103	99%	5040/5080	99%	
Overall study popuation	109/5068	2.2%	160/5040	3.2%	RR 0.68, 95% CI 0.55-0.84, p=0.002
Grade 1 hypertension	64/3591	1.8%	82/3559	2.3%	RR 0.78, 95% CI 0.57-1.06, p=0.10
Grade 2 hypertension	34/1084	3.1%	50/1028	4.9%	RR 0.64, 95% CI 0.42-0.98, p=0.041
Grade 3 hypertension	11/393	2.8%	28/453	6.2%	RR 0.45, 95% CI 0.26-0.79, p=0.009

Tabulation of vital status assessment and mortality among individual participants, excluding adults ≥80 years of age. Baseline hypertension severity defined by lowest of three blood pressure (BP) measurements and classified as not hypertensive (<140/90 mmHg), Grade 1 (BP 140-159/90-99 mmHg), Grade 2 (BP 160-179/100-109 mmHg), Grade 3 (BP ≥180/110 mmHg). TMLE, targeted maximum likelihood estimation.

Table G. Mortality by year three among HIV-uninfected adults with baseline uncontrolled hypertension

	Intervention		Control		Effect Estimate (calculated at cluster level using TMLE, see methods)
	n	%	n	%	
Vital status assessed	5069/5102	99%	5113/5149	99%	
Overall study popuation	162/5069	3.2%	208/5113	4.1%	RR 0.80, 95% CI 0.64-0.98, p=0.037
Grade 1 hypertension	91/3520	2.6%	106/3513	3.0%	RR 0.86, 95% CI 0.64-1.15, p=0.29
Grade 2 hypertension	50/1121	4.5%	61/1096	5.6%	RR 0.82, 95% CI 0.59-1.12, p=0.19
Grade 3 hypertension	21/428	4.9%	41/504	8.1%	RR 0.60, 95% CI 0.39-0.93, p=0.026

Tabulation of vital status assessment and mortality among individual participants, excluding baseline HIV-infected adults. Baseline hypertension severity defined by lowest of three blood pressure (BP) measurements and classified as not hypertensive (<140/90 mmHg), Grade 1 (BP 140-159/90-99 mmHg), Grade 2 (BP 160-179/100-109 mmHg), Grade 3 (BP ≥180/110 mmHg). TMLE, targeted maximum likelihood estimation.

Table H. Characteristics of participants with measured year 3 blood pressure

	**Overall population** n=10,928		**BP measured at year 3** n=8,286	
	n	%	n	%
Region				
Eastern Uganda	4253	39%	3262	39%
Kenya	2786	25%	2031	25%
Western Uganda	3889	36%	2993	36%
Sex				
Female	6058	55%	4763	57%
Male	4870	45%	3523	43%
Age category				
18-29 yrs	1309	12%	751	9%
30-44 yrs	2312	21%	1762	21%
45-59 yrs	3117	29%	2526	30%
60-74 yrs	2888	26%	2351	28%
≥75 yrs	1302	12%	896	11%
Wealth quintile categories*				
First, indicating least wealth	1966	18%	1391	17%
Second	1884	17%	1467	18%
Third	2174	20%	1683	20%
Fourth	2383	22%	1866	23%
Fifth, indicating most wealth	2440	22%	1839	22%
Missing	65	1%	58	1%
Self-reported prior HTN diagnosis	1460	13%	1098	13%
Self-reported baseline HTN treatment	777	7%	574	7%
Baseline hypertension severity†				
Grade 1 (140-159 / 90-99 mmHg)	7585	69%	5768	70%
Grade 2 (160-179 / 100-109 mmHg)	2359	22%	1786	22%
Grade 3 (≥180/110 mmHg)	984	9%	732	9%
Baseline BMI category‡				
Underweight	1643	15%	1220	15%
Healthy weight	6493	59%	4909	59%
Overweight	1986	18%	1541	19%
Obese	716	7%	552	7%
Missing	47	0%	14	0%
Comorbid conditions				
HIV	677	6%	454	5%
Diabetes§	477	4%	350	4%

Characteristics of nonpregnant adults with baseline uncontrolled hypertension who had measured year 3 blood pressure (BP) at population-level testing. *Quintiles calculated using principle-component analysis of the household wealth survey and were calculated at the level of the household. †Baseline hypertension severity defined by lowest of three BP measurements and classified as Grade 1 (BP 140-159/90-99 mmHg), Grade 2 (BP 160-179/100-109 mmHg), Grade 3 (BP ≥180/110 mmHg). ‡Body mass index (BMI) categories include underweight (BMI <18.5 kg/m^2), normal (BMI 18.5-24.9 kg/m^2), overweight (BMI 25-29.9 kg/m^2), or obese (BMI ≥30 kg/m.

Table I. Year three hypertension control, by baseline hypertension severity

	Intervention		Control		Effect Estimate (calculated at cluster level using TMLE, see methods)
	n	%	n	%	
Year 3 blood pressure measured	4173/5459	76%	4113/5459	75%	
Overall study popuation	2200/4173	53%	1764/4113	43%	RR 1.22, 95% CI 1.12-1.33, p=0.0002
Grade 1 hypertension	1752/2924	60%	1475/2844	52%	RR 1.15, 95% CI 1.09-1.22, p=0.0001
Grade 2 hypertension	351/906	39%	227/880	26%	RR 1.50, 95% CI 1.24-1.82, p=0.0004
Grade 3 hypertension	97/343	28%	62/389	16%	RR 1.87, 95% CI 1.13-3.10, p=0.018

Tabulation of year three blood pressure measurement and control, stratified by baseline hypertension severity. Baseline hypertension severity defined by lowest of three blood pressure (BP) measurements and classified as not hypertensive (<140/90 mmHg), Grade 1 (BP 140-159/90-99 mmHg), Grade 2 (BP 160-179/100-109 mmHg), Grade 3 (BP ≥180/110 mmHg). TMLE, targeted maximum likelihood estimation.

Table J. Change in blood pressure from baseline to study year three

	Intervention<br>*Median BP (25th-75th quantile)*	Control<br>*Median BP (25th-75th quantile)*	p-value*
Overall			
Baseline SBP	149 (142 to 161)	150 (143 to 162)	0.09
Year 3 SBP	138 (129 to 150)	141 (132 to 156)	0.0001
Change	-12 (-24 to -1)	-9 (-20 to 3)	0.0001
Baseline Grade 1 HTN			
Baseline SBP	145 (141 to 150)	145 (141 to 151)	0.09
Year 3 SBP	136 (128 to 145)	138 (130 to 149)	0.0004
Change	-9 (-18 to 1)	-7 (-16 to 4)	0.001
Baseline Grade 2 HTN			
Baseline SBP	165 (161 to 171)	166 (162 to 171)	0.07
Year 3 SBP	142 (133 to 157)	151 (138 to 167)	0.001
Change	-21 (-33 to -8)	-14 (-27 to 0)	0.0008
Baseline Grade 3 HTN			
Baseline SBP	189 (182 to 199)	191 (183 to 201)	0.02
Year 3 SBP	152 (137 to 171)	168 (149 to 186)	0.0008
Change	-37 (-54 to -19)	-24 (-46 to -5)	0.002

BP, blood pressure; SBP systolic blood pressure; HTN, hypertension. Blood pressure in mmHg. Baseline grade 1 hypertension defined as lowest baseline blood pressure 140-159/90-99 mmHg, grade 2 HTN defined as baseline blood pressure 160-179/100-109 mmHg, and grade 3 HTN defined as baseline blood pressure ≥180/110 mmHg. *Post-hoc comparisons by trial arm calculated with cluster-level paired t-test.

Table K. Baseline characteristics of participants who died by study year three

	Intervention	Control
Overall	174/5422 (3.2%)	225/5426 (4.1%)
Region		
Eastern Uganda	41/2110 (1.9%)	69/2118 (3.3%)
Kenya	68/1337 (5.1%)	85/1425 (6%)
Western Uganda	65/1975 (3.3%)	71/1883 (3.8%)
Sex		
Female	89/3021 (2.9%)	110/3004 (3.7%)
Male	85/2401 (3.5%)	115/2422 (4.7%)
Age category		
18-29 yrs	3/619 (0.5%)	7/667 (1%)
30-44 yrs	14/1184 (1.2%)	14/1102 (1.3%)
45-59 yrs	25/1541 (1.6%)	45/1559 (2.9%)
60-74 yrs	52/1435 (3.6%)	64/1444 (4.4%)
≥75 yrs	80/643 (12.4%)	95/654 (14.5%)
Wealth quintile categories*		
First, indicating least wealth	39/926 (4.2%)	58/1028 (5.6%)
Second	32/899 (3.6%)	37/980 (3.8%)
Third	42/1088 (3.9%)	42/1085 (3.9%)
Fourth	31/1176 (2.6%)	48/1199 (4%)
Fifth, indicating most wealth	30/1319 (2.3%)	38/1100 (3.5%)
Missing	0/14 (0%)	2/34 (5.9%)
Self-reported prior HTN diagnosis	31/688 (4.5%)	43/765 (5.6%)
Self-reported baseline HTN treatment	20/363 (5.5%)	29/410 (7.1%)
Baseline hypertension severity†		
Grade 1 (140-159 / 90-99 mmHg)	98/3783 (2.6%)	112/3742 (3%)
Grade 2 (160-179 / 100-109 mmHg)	54/1188 (4.5%)	71/1157 (6.1%)
Grade 3 (≥180/110 mmHg)	22/451 (4.9%)	42/527 (8%)
Baseline BMI category‡		
Underweight	63/828 (7.6%)	57/803 (7.1%)
Healthy weight	88/3268 (2.7%)	124/3176 (3.9%)
Overweight	18/941 (1.9%)	33/1033 (3.2%)
Obese	5/366 (1.4%)	7/345 (2%)
Missing	0/19 (0%)	4/69 (5.8%)
Comorbid conditions		
HIV	12/353 (3.4%)	17/313 (5.4%)
Diabetes	17/225 (7.6%)	17/247 (6.9%)

Characteristics of nonpregnant adults with baseline uncontrolled hypertension who died by study year three. *Quintiles calculated using principle-component analysis of the household wealth survey and were calculated at the level of the household. †Baseline hypertension severity defined by lowest of three blood pressure (BP) measurements and classified as Grade 1 (BP 140-159/90-99 mmHg), Grade 2 (BP 160-179/100-109 mmHg), Grade 3 (BP ≥180/110 mmHg). ‡Body mass index (BMI) categories include underweight (BMI <18.5 kg/m^2), normal (BMI 18.5-24.9 kg/m^2), overweight (BMI 25-29.9 kg/m^2), or obese (BMI ≥30 kg/m^2).

Table L. Baseline characteristics of participants with controlled hypertension at study year three

	Intervention	Control
Overall	2200/4173 (53%)	1764/4113 (43%)
Region		
Eastern Uganda	826/1632 (51%)	625/1630 (38%)
Kenya	568/971 (58%)	542/1060 (51%)
Western Uganda	806/1570 (51%)	597/1423 (42%)
Sex		
Female	1228/2416 (51%)	952/2347 (41%)
Male	972/1757 (55%)	812/1766 (46%)
Age category		
18-29 yrs	252/353 (71%)	251/398 (63%)
30-44 yrs	512/917 (56%)	405/845 (48%)
45-59 yrs	622/1267 (49%)	477/1259 (38%)
60-74 yrs	605/1185 (51%)	448/1166 (38%)
≥75 yrs	209/451 (46%)	183/445 (41%)
Wealth quintile categories*		
First, indicating least wealth	386/673 (57%)	337/718 (47%)
Second	382/704 (54%)	314/763 (41%)
Third	440/849 (52%)	341/834 (41%)
Fourth	482/936 (51%)	410/930 (44%)
Fifth, indicating most wealth	502/999 (50%)	349/840 (42%)
Missing	8/12 (67%)	13/28 (46%)
Self-reported prior HTN diagnosis	221/534 (41%)	169/564 (30%)
Self-reported baseline HTN treatment	119/277 (43%)	85/297 (29%)
Baseline hypertension severity†		
Grade 1 (140-159 / 90-99 mmHg)	1752/2924 (60%)	1475/2844 (52%)
Grade 2 (160-179 / 100-109 mmHg)	351/906 (39%)	227/880 (26%)
Grade 3 (≥180/110 mmHg)	97/343 (28%)	62/389 (16%)
Baseline BMI category‡		
Underweight	359/614 (58%)	274/606 (45%)
Healthy weight	1346/2516 (53%)	1075/2393 (45%)
Overweight	360/747 (48%)	315/794 (40%)
Obese	130/282 (46%)	86/270 (32%)
Missing	5/14 (36%)	14/50 (28%)
Comorbid conditions		
HIV	159/240 (66%)	119/214 (56%)
Diabetes	77/160 (48%)	53/190 (28%)

Characteristics of participants with baseline uncontrolled hypertension who had controlled blood pressure at year three community health campaign. *Quintiles calculated using principle-component analysis of the household wealth survey and were calculated at the level of the household. †Baseline hypertension severity defined by lowest of three blood pressure (BP) measurements and classified as Grade 1 (BP 140-159/90-99 mmHg), Grade 2 (BP 160-179/100-109 mmHg), Grade 3 (BP ≥180/110 mmHg). ‡Body mass index (BMI) categories include underweight (BMI <18.5 kg/m^2), normal (BMI 18.5-24.9 kg/m^2), overweight (BMI 25-29.9 kg/m^2), or obese (BMI ≥30 kg/m^2).

Table M. Baseline characteristics of participants who linked to care within one year of baseline hypertension screening (Uganda only)

	Intervention	Control
Overall	1810/4112 (44%)	1413/4030 (35%)
Region		
Eastern Uganda	806/2117 (38%)	632/2136 (30%)
Western Uganda	1004/1995 (50%)	781/1894 (41%)
Sex		
Female	1035/2179 (47%)	817/2141 (38%)
Male	775/1933 (40%)	596/1889 (32%)
Age category		
18-29 yrs	101/493 (20%)	86/512 (17%)
30-44 yrs	377/986 (38%)	266/907 (29%)
45-59 yrs	583/1192 (49%)	456/1206 (38%)
60-74 yrs	531/985 (54%)	428/968 (44%)
≥75 yrs	218/456 (48%)	177/437 (41%)
Wealth quintile categories*		
First, indicating least wealth	353/801 (44%)	303/887 (34%)
Second	310/764 (41%)	275/830 (33%)
Third	371/848 (44%)	301/816 (37%)
Fourth	390/865 (45%)	274/808 (34%)
Fifth, indicating most wealth	360/806 (45%)	228/652 (35%)
Missing	26/28 (93%)	32/37 (86%)
Self-reported prior HTN diagnosis	320/537 (60%)	283/632 (45%)
Self-reported baseline HTN treatment	180/285 (63%)	164/344 (48%)
Baseline hypertension severity†		
Grade 1 (140-159 / 90-99 mmHg)	1139/2898 (39%)	795/2788 (29%)
Grade 2 (160-179 / 100-109 mmHg)	473/887 (53%)	391/847 (46%)
Grade 3 (≥180/110 mmHg)	198/327 (61%)	227/395 (57%)
Baseline BMI category‡		
Underweight	264/642 (41%)	230/627 (37%)
Healthy weight	1047/2469 (42%)	774/2335 (33%)
Overweight	361/719 (50%)	294/771 (38%)
Obese	133/269 (49%)	106/263 (40%)
Missing	5/13 (38%)	9/34 (26%)
Comorbid conditions		
HIV	159/174 (91%)	113/180 (63%)
Diabetes	123/203 (61%)	109/215 (51%)

Characteristics of participants with baseline uncontrolled hypertension in Uganda who linked to hypertension care within one year of baseline screening. *Quintiles calculated using principle-component analysis of the household wealth survey and were calculated at the level of the household. †Baseline hypertension severity defined by lowest of three blood pressure (BP) measurements and classified as Grade 1 (BP 140-159/90-99 mmHg), Grade 2 (BP 160-179/100-109 mmHg), Grade 3 (BP ≥180/110 mmHg). ‡Body mass index (BMI) categories include underweight (BMI <18.5 kg/m^2), normal (BMI 18.5-24.9 kg/m^2), overweight (BMI 25-29.9 kg/m^2), or obese (BMI ≥30 kg/m^2).
