# Supplementary material for: Effect of a patient-centered hypertension delivery strategy on all-cause mortality: Secondary analysis of SEARCH, a community-randomized trial in rural Kenya and Uganda
Source: PLoS Med. 2021 Sep 20;18(9):e1003803. doi: 10.1371/journal.pmed.1003803 (PMC8489716; doi:10.1371/journal.pmed.1003803)
Supplement: S1 Figs — Fig A. Mortality by year 3, among adults <80 years of age. Fig B. Mortality by year 3, among HIV-uninfected adults. Fig C. Mortality by year 3, stratified by sex. Fig D. Mortality by year 3, stratified by country. Fig E. Year 3 hypertension control, stratified by sex. Fig F. Year 3 hypertension control, stratified by country. Fig G. Linkage to hypertension care in Uganda, stratified by sex. Fig H. Care engagement among those linked to care in Uganda. Fig I. Predictors of year 3 hypertension control. Fig J. Predictors of linkage to care (Uganda only). (PDF) [file pmed.1003803.s006.pdf]

S1 Figures. Supplemental Figures

Figure A. Mortality by year three, among adults <80 years of age 2

Figure B. Mortality by year three, among HIV-uninfected adults 3

Figure C. Mortality by year three, stratified by sex 4

Figure D. Mortality by year three, stratified by country 5

Figure E. Year three hypertension control, stratified by sex 6

Figure F. Year three hypertension control, stratified by country 7

Figure G. Linkage to hypertension care in Uganda, stratified by sex 8

Figure H. Care engagement among those linked to care in Uganda 9

Figure I. Predictors of year three hypertension control 10

Figure J. Predictors of linkage to care (Uganda only) 11

Figure A. Mortality by year three, among adults <80 years of age

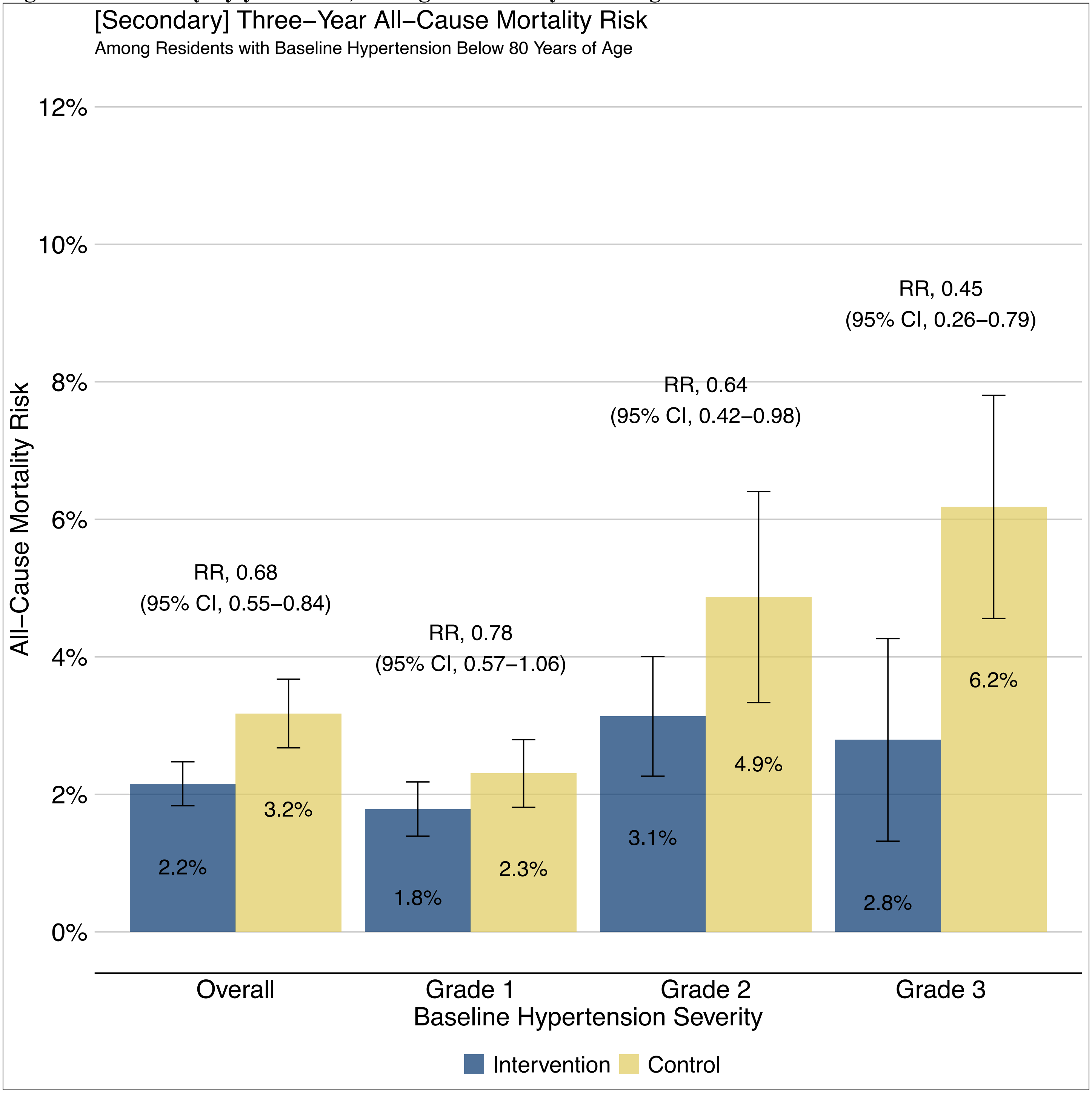

Sensitivity analysis excluding adults 80 years of age or older, in whom hypertension treatment may be deferred due to frailty. Estimates obtained using two-stage targeted maximum likelihood estimation (TMLE) to estimate and compare community-level mortality by three years. Vertical error bars depict arm-specific 95% confidence intervals. Baseline hypertension severity defined by lowest of three blood pressure (BP) measurements and classified as Grade 1 (BP 140-159/90-99 mmHg), Grade 2 (BP 160-179/100-109 mmHg), Grade 3 (BP ≥180/110 mmHg).

Figure B. Mortality by year three, among HIV-uninfected adults

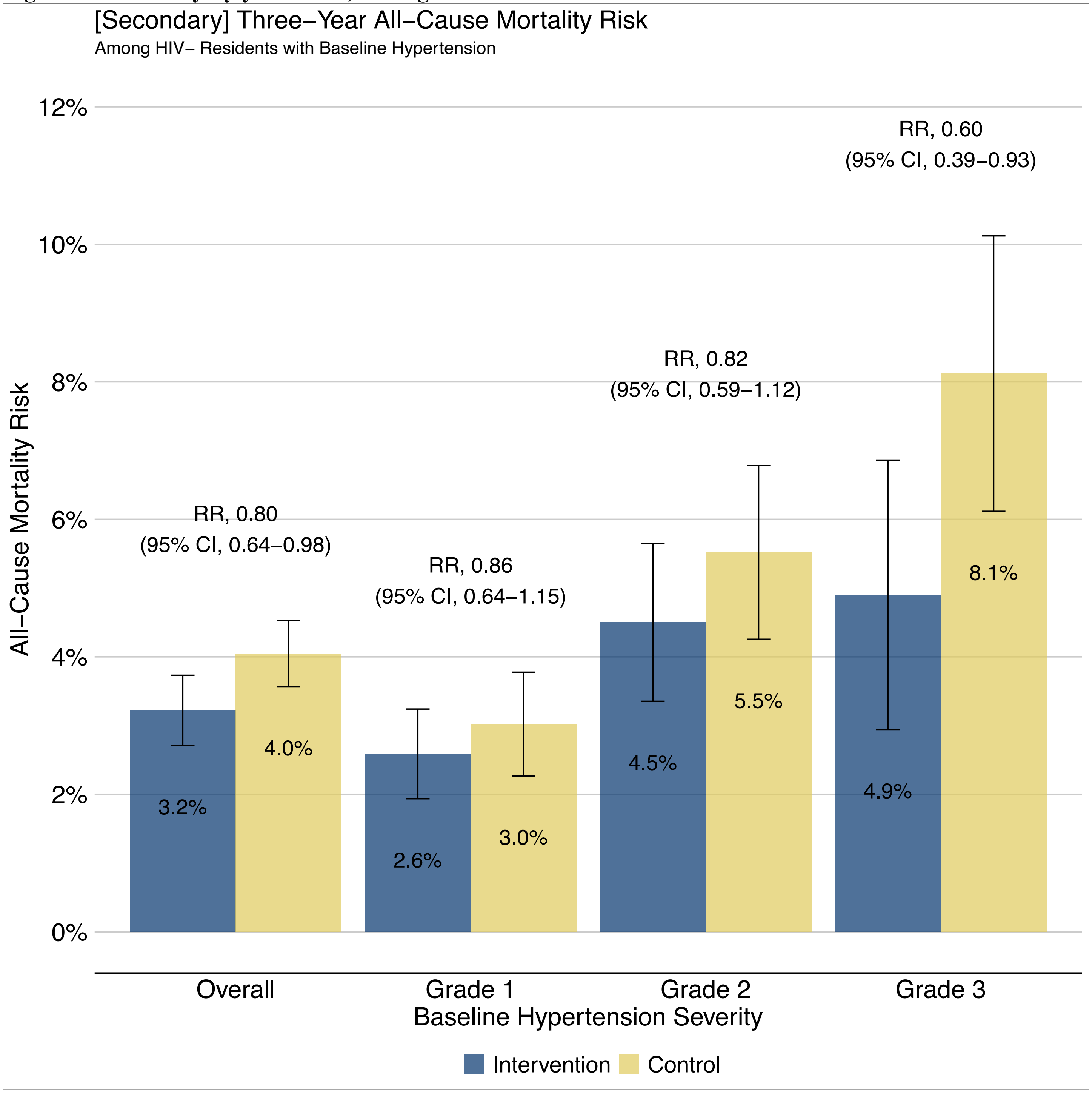

Sensitivity analysis excluding HIV-infected adults to evaluate intervention effect independent from effects of improved HIV care on mortality. Estimates obtained using two-stage targeted maximum likelihood estimation (TMLE) to estimate and compare community-level mortality by three years. Vertical error bars depict arm-specific 95% confidence intervals. Baseline hypertension severity defined by lowest of three blood pressure (BP) measurements and classified as Grade 1 (BP 140-159/90-99 mmHg), Grade 2 (BP 160-179/100-109 mmHg), Grade 3 (BP ≥180/110 mmHg).

Figure C. Mortality by year three, stratified by sex

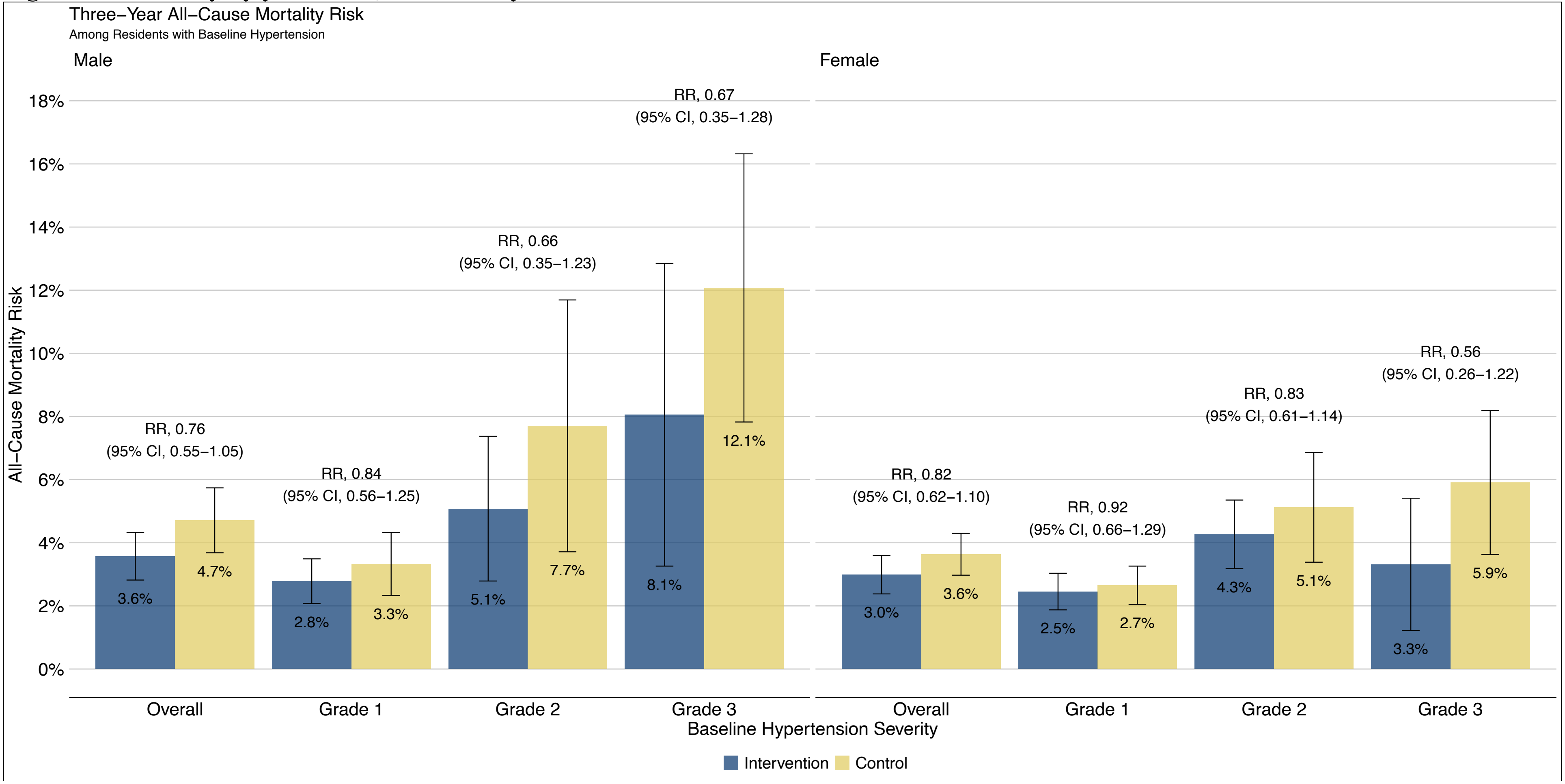

Estimates obtained using two-stage targeted maximum likelihood estimation (TMLE) to estimate and compare community-level mortality by three years. Vertical error bars depict arm-specific 95% confidence intervals. Baseline hypertension severity defined by lowest of three blood pressure (BP) measurements and classified as Grade 1 (BP 140-159/90-99 mmHg), Grade 2 (BP 160-179/100-109 mmHg), Grade 3 (BP ≥180/110 mmHg). Post-hoc analysis for heterogeneity of intervention effect size by sex: p= 0.70 (overall), p=0.65 (Grade 1), p=0.59 (Grade 2), p=0.73 (Grade 3).

Figure D. Mortality by year three, stratified by country

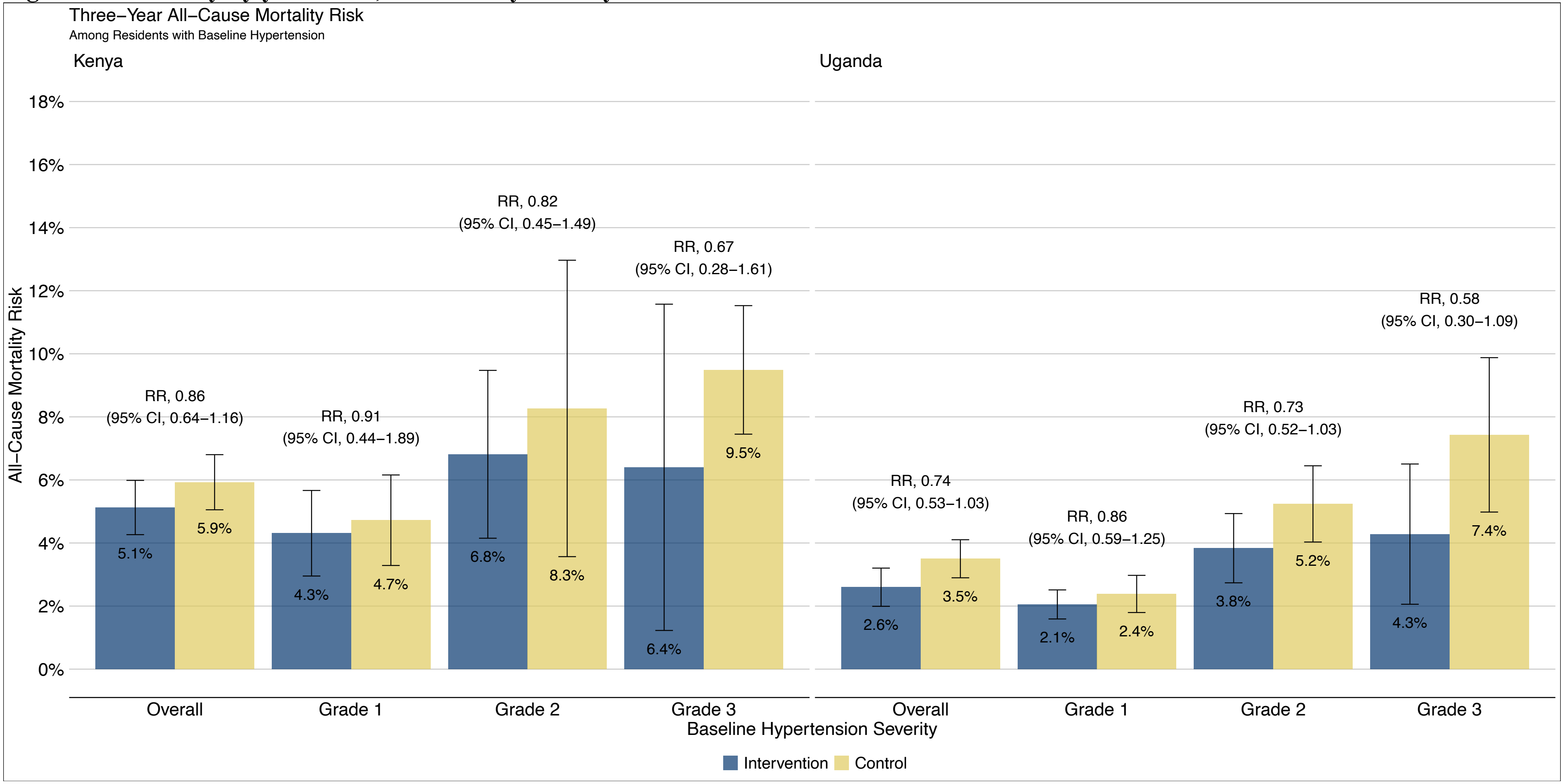

Post-hoc analysis stratifying intervention effect on mortality by country (n=12 communities in Kenya, n=20 communities in Uganda). Estimates obtained using two-stage targeted maximum likelihood estimation (TMLE) to estimate and compare community-level mortality by three years. Vertical error bars depict arm-specific 95% confidence intervals. Baseline hypertension severity defined by lowest of three blood pressure (BP) measurements and classified as Grade 1 (BP 140-159/90-99 mmHg), Grade 2 (BP 160-179/100-109 mmHg), Grade 3 (BP ≥180/110 mmHg). Post-hoc analysis for heterogeneity of intervention effect size by country: p= 0.41 (overall), p=0.57 (Grade 1), p=0.88 (Grade 2), p=0.94 (Grade 3).

Figure E. Year three hypertension control, stratified by sex

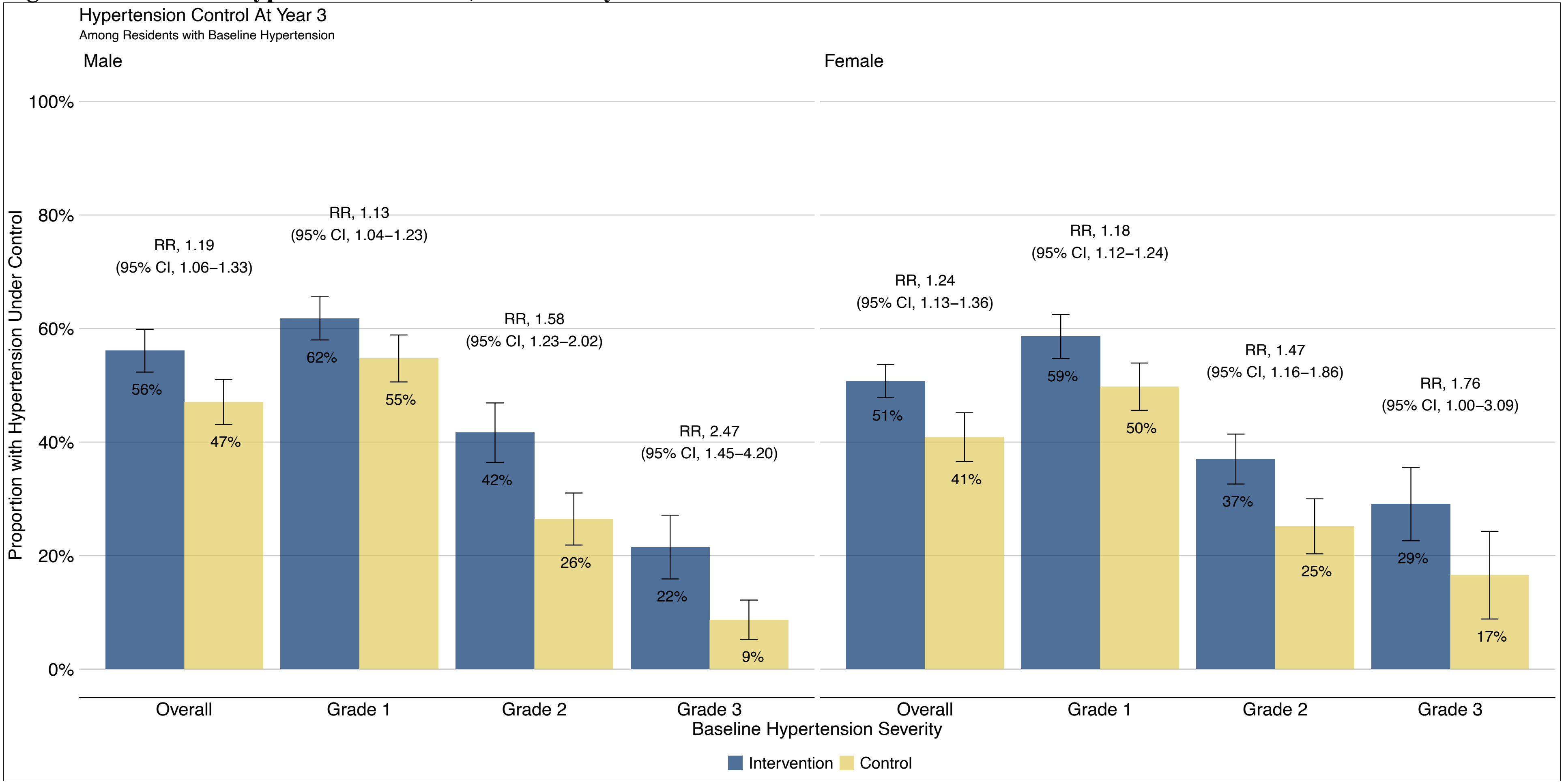

Estimates obtained using two-stage targeted maximum likelihood estimation (TMLE) to estimate and compare community-level hypertension control at year three population-level blood pressure measurement. Vertical error bars depict arm-specific 95% confidence intervals. Hypertension control defined as the lowest of three blood pressure measurements <140/90 mmHg. Baseline hypertension severity defined by lowest of three blood pressure (BP) measurements and classified as Grade 1 (BP 140-159/90-99 mmHg), Grade 2 (BP 160-179/100-109 mmHg), Grade 3 (BP ≥180/110 mmHg). Post-hoc analysis for heterogeneity of intervention effect size by sex: p= 0.51 (overall), p=0.43 (Grade 1), p=0.64 (Grade 2), p=0.23 (Grade 3).

Figure F. Year three hypertension control, stratified by country

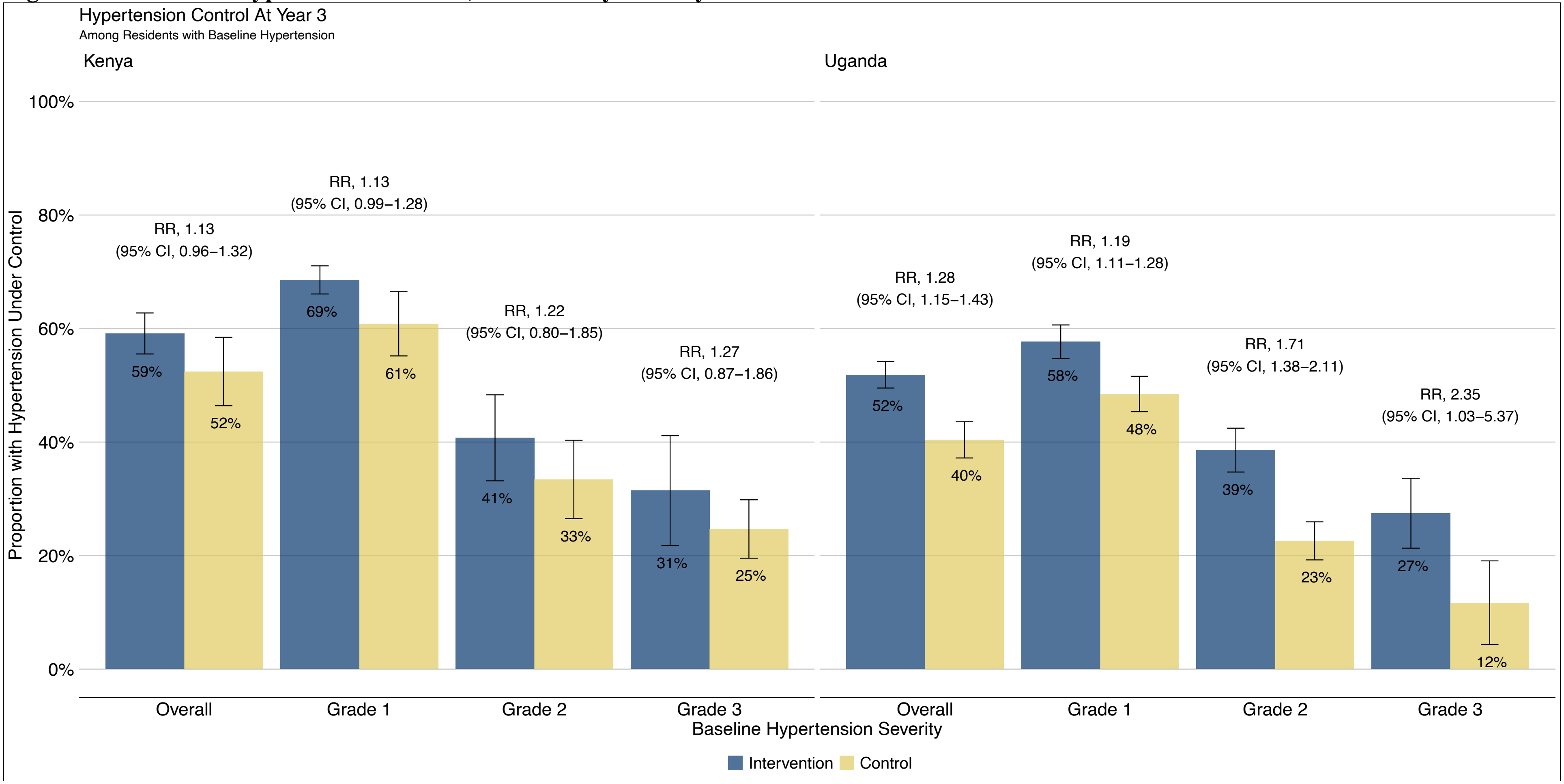

Post-hoc analysis stratifying intervention effect on year three hypertension control by country (n=12 communities in Kenya, n=20 communities in Uganda). Estimates obtained using two-stage targeted maximum likelihood estimation (TMLE) to estimate and compare community-level hypertension control at year three population-level blood pressure measurement. Vertical error bars depict arm-specific 95% confidence intervals. Hypertension control defined as the lowest of three blood pressure measurements <140/90 mmHg. Baseline hypertension severity defined by lowest of three blood pressure (BP) measurements and classified as Grade 1 (BP 140-159/90-99 mmHg), Grade 2 (BP 160-179/100-109 mmHg), Grade 3 (BP ≥180/110 mmHg). Post-hoc analysis for heterogeneity of intervention effect size by country: p= 0.04 (overall), p=0.23 (Grade 1), p=0.02 (Grade 2), p=0.06 (Grade 3).

Figure G. Linkage to hypertension care in Uganda, stratified by sex

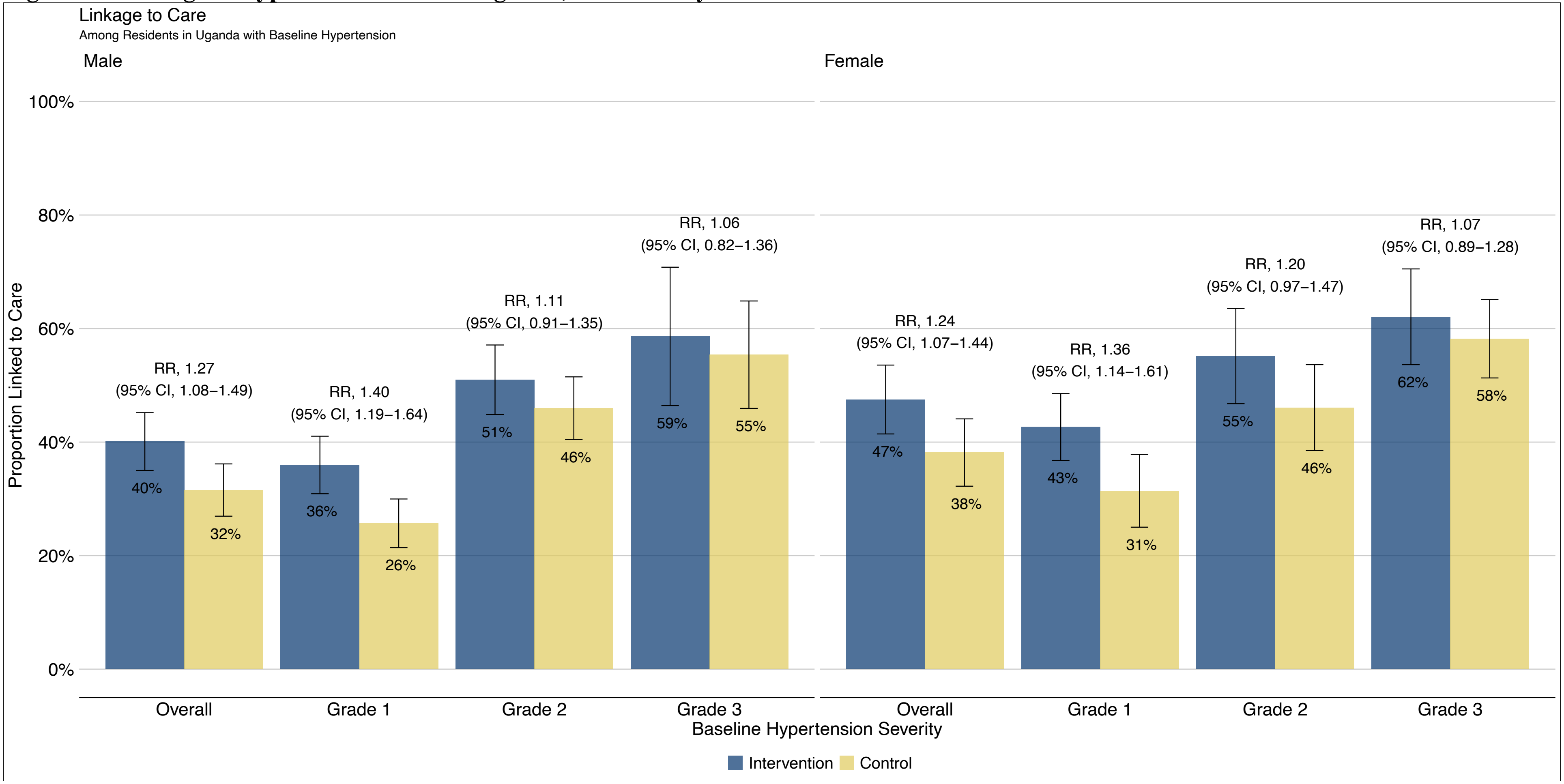

Estimates obtained using two-stage targeted maximum likelihood estimation (TMLE) to estimate and compare the community-level proportion linking to care in Uganda. Vertical error bars depict arm-specific 95% confidence intervals. Linkage to care defined as ≥1 clinic visit for hypertension care within the first year following baseline hypertension screening. Baseline hypertension severity defined by lowest of three blood pressure (BP) measurements and classified as Grade 1 (BP 140-159/90-99 mmHg), Grade 2 (BP 160-179/100-109 mmHg), Grade 3 (BP ≥180/110 mmHg). Post-hoc analysis for heterogeneity of intervention effect size by sex: p= 0.77 (overall), p=0.63 (Grade 1), p=0.56 (Grade 2), p=0.96 (Grade 3).

Figure H. Care engagement among those linked to care in Uganda

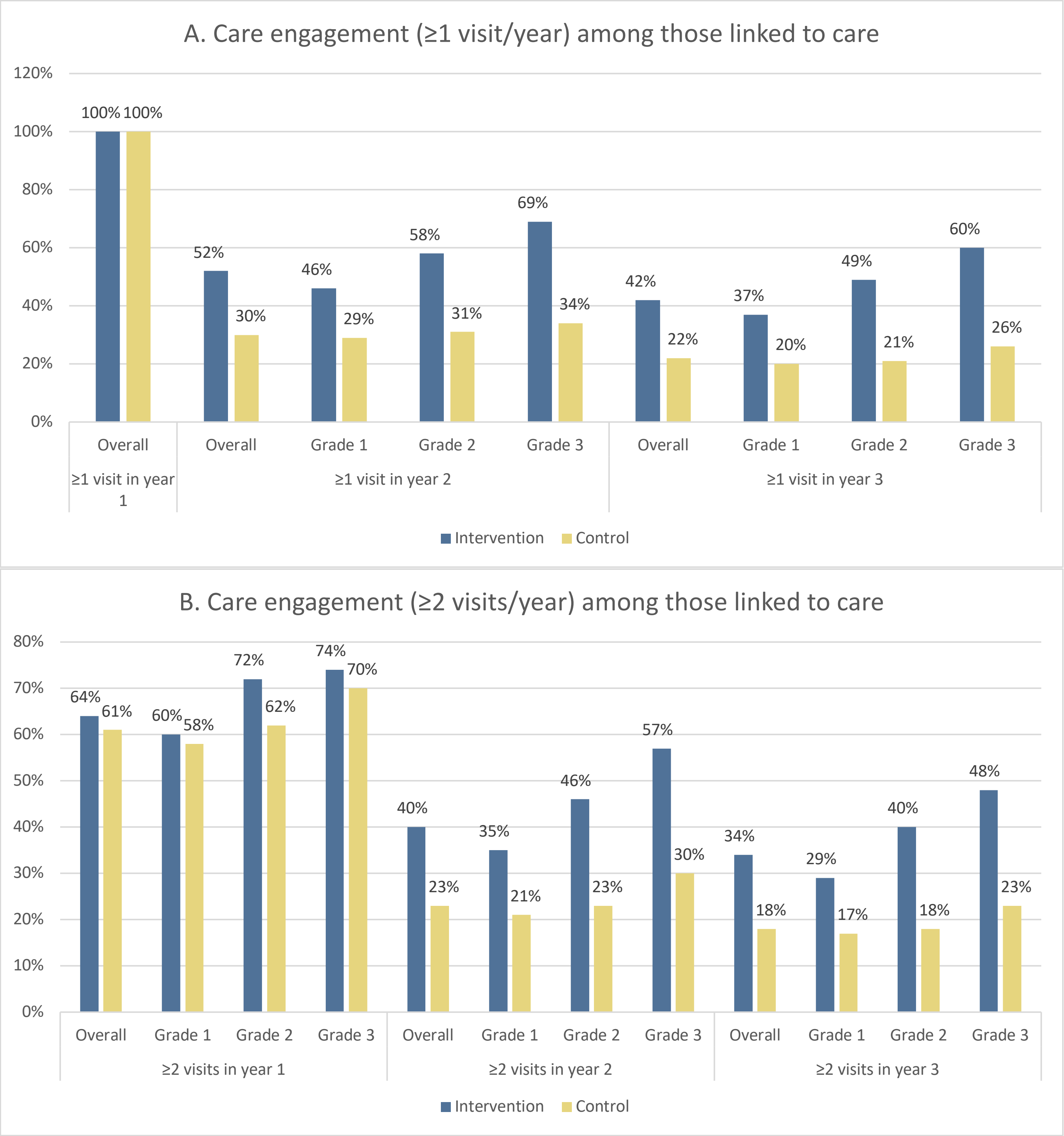

For both panels, individual patient tabulation of hypertension visits by study year (not assessed using community-level TMLE). Linkage defined as at least one clinic visit for hypertension care in the first year following baseline hypertension screening. Baseline hypertension severity defined by lowest of three blood pressure (BP) measurements and classified as Grade 1 (BP 140-159/90-99 mmHg), Grade 2 (BP 160-179/100-109 mmHg), Grade 3 (BP ≥180/110 mmHg). Panel A: Care engagement defined as at least one visit for hypertension care in each year, conditional on having a visit in the prior year. Panel B: Care engagement defined as at least two visits for hypertension care each year, conditional on meeting engagement criteria in the prior year.

Figure I. Predictors of year three hypertension control

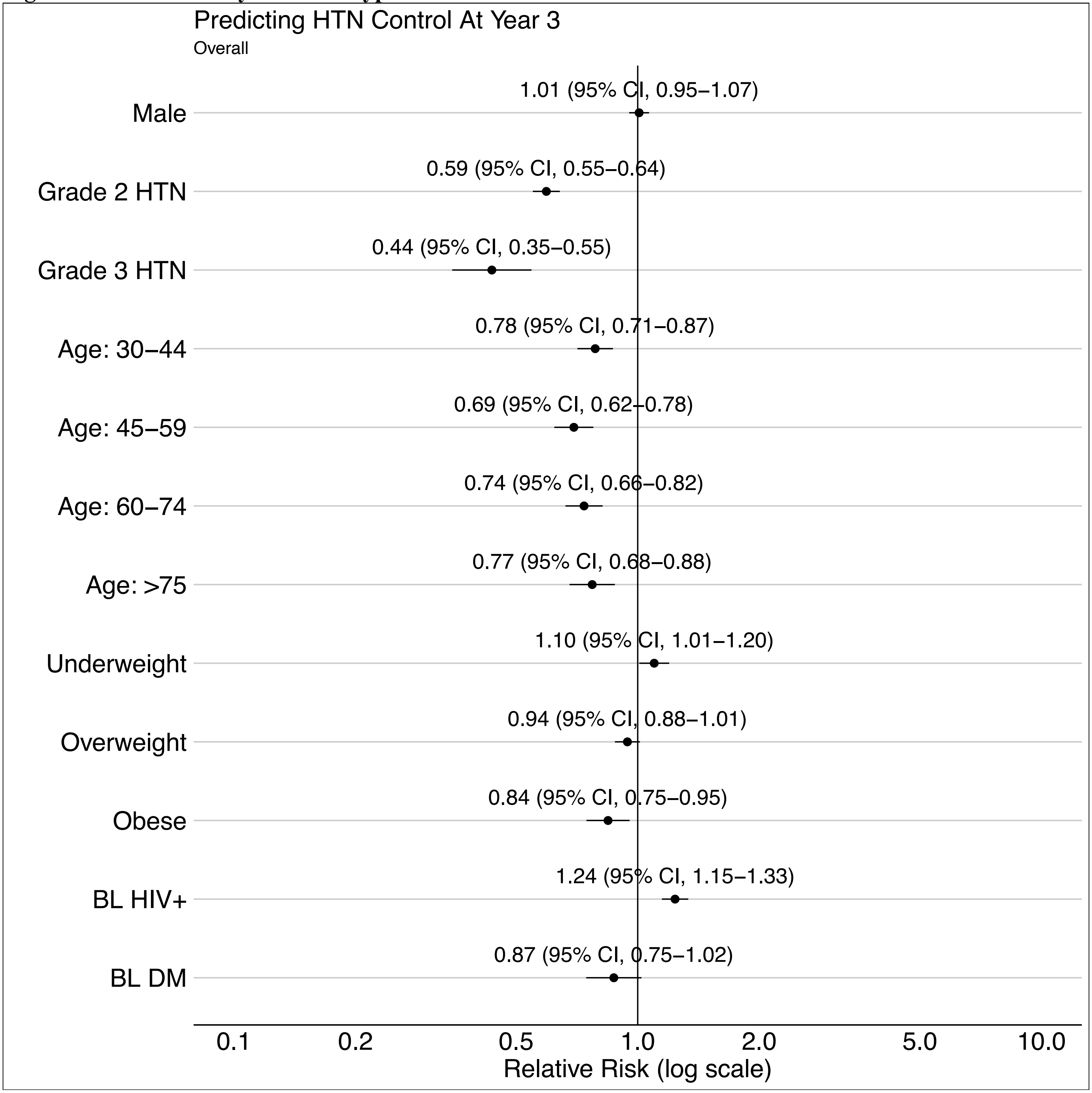

HTN, hypertension; BL, baseline; DM, diabetes mellitus. Predictors of hypertension control by study year three using multivariable TMLE, with relative risks for each variable compared to reference value. Horizontal error bars depict predictor-specific 95% confidence intervals. Reference values for relevant categorical variables include female, grade 1 hypertension, age 18-29 years, normal body mass index, baseline HIV uninfected, and baseline absence of diabetes. Baseline hypertension severity defined by lowest of three blood pressure (BP) measurements and classified as Grade 1 (BP 140-159/90-99 mmHg), Grade 2 (BP 160-179/100-109 mmHg), Grade 3 (BP ≥180/110 mmHg). Body mass index (BMI) categories include underweight (BMI <18.5 kg/m^2), normal (BMI 18.5-24.9 kg/m^2), overweight (BMI 25-29.9 kg/m^2), or obese (BMI ≥30 kg/m^2).

Figure J. Predictors of linkage to care (Uganda only)

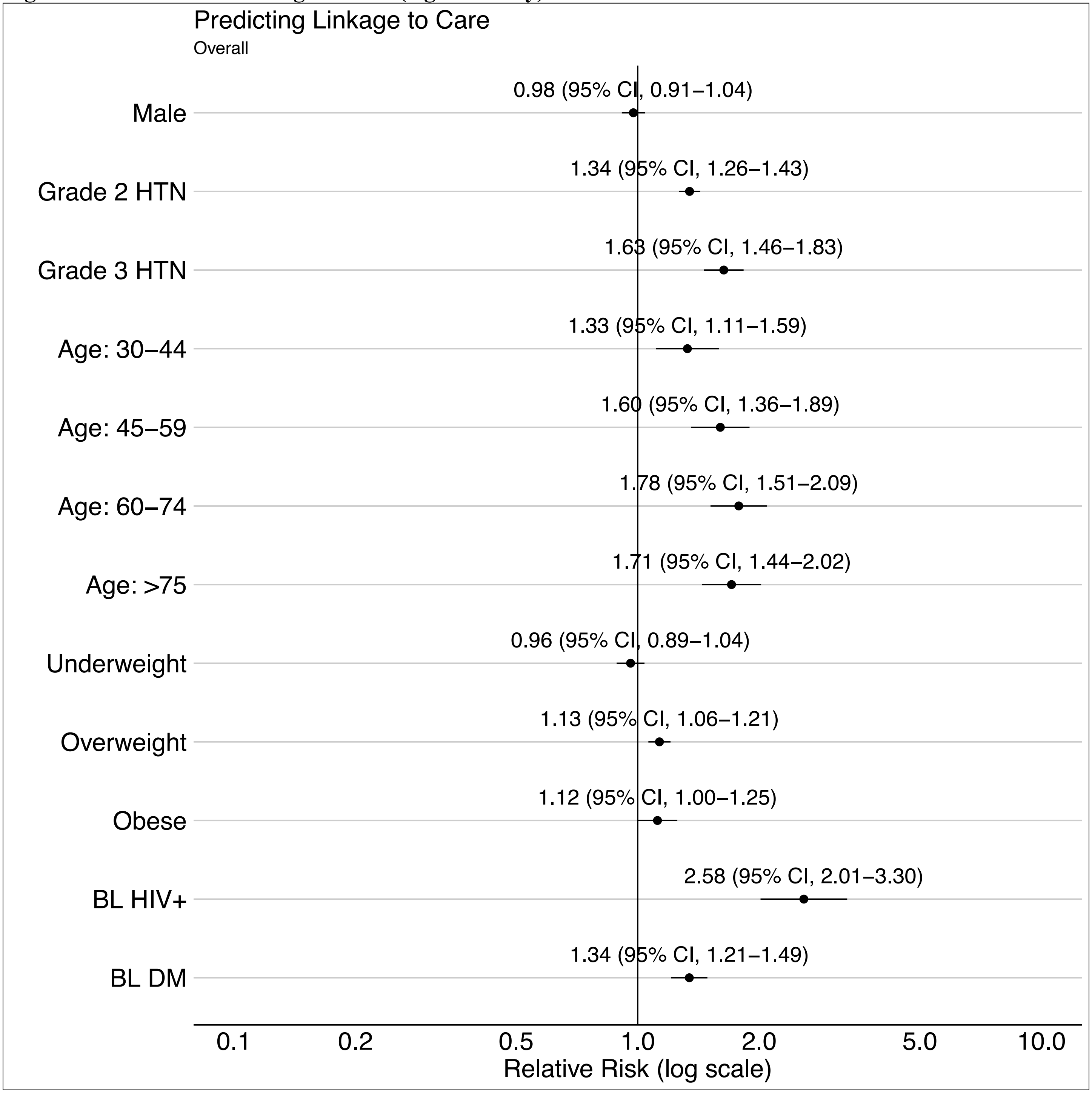

HTN, hypertension; BL, baseline; DM, diabetes mellitus. Predictors of linkage to care in Uganda within one year of baseline screening using multivariable TMLE, with relative risks for each variable compared to reference value. Horizontal error bars depict predictor-specific 95% confidence intervals. Reference values for relevant categorical variables include female, grade 1 hypertension, age 18-29 years, normal body mass index, baseline HIV uninfected, and baseline absence of diabetes. Baseline hypertension severity defined by lowest of three blood pressure (BP) measurements and classified as Grade 1 (BP 140-159/90-99 mmHg), Grade 2 (BP 160-179/100-109 mmHg), Grade 3 (BP ≥180/110 mmHg). Body mass index (BMI) categories include underweight (BMI <18.5 kg/m^2), normal (BMI 18.5-24.9 kg/m^2), overweight (BMI 25-29.9 kg/m^2), or obese (BMI ≥30 kg/m^2).
